# Supplementary material for: A supervised multiclass framework for mineral classification of Iberian beads
Source: PLoS One. 2024 Jul 10;19(7):e0302563. doi: 10.1371/journal.pone.0302563 (PMC11236108; doi:10.1371/journal.pone.0302563)
Supplement: S1 Appendix — PDF document with specific information about sample location, feature description and tab captions. (PDF) [file pone.0302563.s002.pdf]

Supplementary information

Dataset Description

Daniel Sanchez-Gomez[1*#]; Carlos P. Odriozola[1,2]; Ana C. Sousa[1]; José Ángel Garrido-Cordero[2]; Galo Romero-García[2]; José María Martínez-Blanes[3,4]; Manuel Edo i Benaiges[5]; Rodrigo Villalobos-García[6], Victor S. Gonçalves[1]

Centro de Arqueologia da Universidade de Lisboa (UNIARQ), Lisbon, Portugal.

[2]Dpto. de Prehistoria y Arqueología, Universidad de Sevilla, Seville, Spain.

[3]Instituto de Ciencia de Materiales de Sevilla, Universidad de Sevilla- Consejo Superior de Investigaciones Científicas, Seville, Spain.

[4]Dpto. de Química Inorgánica, Universidad de Sevilla, Seville, Spain.

[5]Institut d'Arqueologia. Universitat de Barcelona, Spain.

[6] Cuerpo de Profesores de Enseñanza Secundaria. Gobierno de Cantabria, Spain.

Description:

This dataset contains the data used for the training of a supervised multiclass framework for the mineral classification of prehistoric personal adornment objects.
The dataset contains data on the chemical and mineral compositions of (n=1243) geoarchaeological samples from prehistoric mining sources and prehistoric sites (n=52 sites) spanning from 5th-2nd millennia BC on the Iberian Peninsula.
The data on the concentrations of chemical elements have been obtained by p-XRF and the labels on the mineral species composing each sample have been obtained by XRD.

The training dataset as well as the data used as proof of concept are fully available as part of the supplementary material to the manuscript.

Source:

The data used in this set come from 52 sites on the Iberian Peninsula and have been obtained in different research, excavation and survey campaigns by different members of the

research team, as well as from visits to different academic centers and museums over a period of more than ten years.[1–10]

The data from samples from Portuguese sites are located in museums:

- Museu Municipal de Torres Novas (Torres Novas, Portugal)
- Museu dos Serviços Geológicos de Portugal (Lisbon, Portugal)

The data from the north-east of the Peninsula come from specimens from the excavations carried out by (MEiB) as well as from the following centers and museums:

- Museu Nacional de Arqueología de Catalunya (Barcelona, Spain)
- Seminari d'Estudis I Recerques Prehistòriques (University of Barcelona, Spain)

The data from the south of the Iberian Peninsula come from the researches of (CO and JAGC) as well as from specimens found in museums:

- Museo de Huelva (Huelva, Spain)
- Departamento de Prehistoria y Arqueología (Universidad de Sevilla, Spain)

The data from the Spanish north and central plateau come from the researches of (CO, RVG, JAG) as well as from specimens in the following museums:

- Museo de Ávila
- Museo de Salamanca
- Museo de Segovia
- Museo de Zamora
- Museo arqueológico provincial de Palencia
- Museo de Santa cruz. Toledo

The proof-of-concept data come from unpublished samples of the researchers (AS and VG).

No human remains were used in the present study.

No permits were required for the analysis of geological samples. Permits for the analyses of archaeological samples were granted by the Chief Curators and Heads of the museums listed above

The dataset includes geographic information for each of the sites included in the study. Therefore, researchers interested in particular pieces can perform a geographic search by site to locate the administrative entity (locality, autonomous community, city council) and corresponding museum.

A repository with our source code and training data, conveniently packaged with README instructions and links for reuse, is publicly available on the GitHub page (https://github.com/Daniel-SanchezG/MACLAS)

Size and feature description:

The file is presented in excel spreadsheet format with the following tabs: XRF_raw, Training, Proof-of-concept, mineral_samples_per_site, Dataset_description, References, Dataset_description, pXRF_LOD, Reference Material and Ref_Material_Stats:

XRF_raw:

This tab contains the XRF raw data before its preprocess. It contains 92 columns and 1244 rows

Feature	Data_type	Feature description
Name	String	Key field: Unique identifier
Duration	Float	Measuring time in seconds
Mg to Th	Float	Elemental concentration per chemical element in wt.% obtained by means of p-XRF
+/-	Float	Standard deviation (precision) reported per each value in the previous column

Training:

This tab contains the data used to train the model. It contains 53 variables and 1244 rows

Feature	Data_type	Feature description
Name	String	Key field: Unique identifier
XRD_MIN	String	Main mineral phase label obtained by means of XRD
XRD_MIN_2	String	Secondary mineral phase label obtained by means of XRD (optional)
XRD_MIN_3	String	Secondary mineral phase label obtained by means of XRD (optional)
XRD_MIN_4	String	Secondary mineral phase label obtained by means of XRD (optional)
Mineral_Species_Target	String	Target variable: manually created mineral species Label
Major_Mineral_Groups_Target	String	Target variable: Major mineral group according to the strunz system
Mg to Th	Float	Predictor variables: atomic percentage of each chemical element obtained by means of p-XRF
Σ	Integer num	Closure of compositional quantities (100%)

Proof-of-concept tab:

Contains the data used to test the model under real-world conditions. It contains 48 columns and 25 rows

Feature	Data_type	Feature description
Name:	String	Key field: Unique identifier
XRD_MIN	String	Ground truth.Main mineral phase label obtained by means of XRD
XRD_MIN_2	String	Ground truth.Secondary mineral phase label obtained by means of XRD (optional)
Mg to Th	Float	Predictor variables: atomic percentage per chemical element obtained by means of p-XRF

Mineral_samples_per_site:

This tab contains contextual information about the distribution of raw material among the sites used in this study. It contains 23 columns and 53 rows:

Feature	Data_type	Feature description
Longitude	Float	geographic coordinates in decimal format Datum: WGS84 1984
Latitude	Float	geographic coordinates in decimal format Datum: WGS84 1985
Chronology	Ordinal	Relative chronology of the sites expressed in millennia
Site	String	Site identifier (G) refers to sites with geological samples only
Variscite	Integer	Number of samples per site
Aheylite	Integer	Number of samples per site
Berlinite	Integer	Number of samples per site
Crandallite	Integer	Number of samples per site
Planerite	Integer	Number of samples per site
Turquoise	Integer	Number of samples per site
Strengite	Integer	Number of samples per site
Annabergite	Integer	Number of samples per site
Metavariscite	Integer	Number of samples per site
Muscovite	Integer	Number of samples per site
Talc	Integer	Number of samples per site
Chlorite-serpentine	Integer	Number of samples per site
Illite	Integer	Number of samples per site
Clinochlore	Integer	Number of samples per site
Fluorite	Integer	Number of samples per site
Calcite	Integer	Number of samples per site

Aragonite	Integer	Number of samples per site
Quartz	Integer	Number of samples per site
total	Integer	Total number of samples used for model development (training, testing and validation)

Dataset_description:

It contains the description of the dataset in tabular format. It is composed of 4 columns and 37 rows recording the same information exposed here.

Feature	Data_type	Feature description
Tab	String	Name of each tab within the dataset
Feature	String	Feature name within each tab
Data_type	String	Data type of each feature
Feature description	String	Description of each feature within the dataset

p-XRF LOD

This tab reports the Limits of Detection (LoD's) of the instrument and the values used to impute below detection limit values.

Feature	Data_type	Feature description
Element	String	Symbol of chemical elements from Mg to Th
Measure time	String	Measurement time for which the Limit of Detection is present (LoD). LOD's improve as a function of the square root of the testing time.
LoD ppm	Integer	LoD is specified for each matrices in three sigma 99.7% confidence level.
LoD at%	Float	Description of each feature within the dataset
LoD/sqrt(2)	Float	Imputation values for BDL

Reference_Material:

This tab contains the raw XRF data of the BCR032 Moroccan Phosphate Reference material. Values are presented in wt%

Feature	Data_type	Feature description
Name	String	Key field: Unique identifier
Class	String	Device configuration for Quantification
Duration	Float	Measuring time in seconds
Mg to Th	Float	Elemental concentration in wt% per chemical element obtained by means of p-XRF
+/-	Float	Standard deviation (precision) reported per each value in the previous column

Ref_Material_Stats:

This tab contains the descriptive statistics of the Reference Material Morocco Phosphate BCR032, comparative information between the reference values and the measurements obtained as well as correction factors for transforming Oxides into elements as the reference values are reported as oxides. The reference values are obtained from https://crm.jrc.ec.europa.eu/p/40455/40468/By-material-matrix/Other-manufactured-materials/BCR-032-MOROCCAN-PHOSPHATE-ROCK-trace-elements/BCR-032

References:

1. Odriozola CP. A new approach to determine the geological provenance of variscite artifacts using the P/Al atomic ratios. Archaeol Anthropol Sci. 2015;7: 329–350. doi:10.1007/s12520-014-0195-2
2. Odriozola CP, Linares-Catela JA, Hurtado-Pérez VM. Provenancing variscite beads: Pico Centeno (Encinasola, Spain) outcrop case study. Open J Archaeometry. 2013;1: 17. doi:10.4081/arc.2013.e17
3. Odriozola CP, García RV, Burbidge CI, Boaventura R, Sousa AC, Rodriguez-Ariza O, et al. Distribution and chronological framework for Iberian variscite mining and consumption at Pico Centeno, Encinasola, Spain. Quaternary Research. 2016;85: 159–176. doi:10.1016/j.yqres.2015.11.010
4. Odriozola CP, Garrido-Cordero JÁ, Santos C, Barradas E, Sousa AC. The stone beads from Barrada’s hypogeum 1 (Aljezur, Algarve, Portugal). Greenstone distribution patterns in the Iberian Southwest late Neolithic. Journal of Archaeological Science: Reports. 2020;34: 102667. doi:10.1016/j.jasrep.2020.102667
5. Garrido-Cordero JÁ, Odriozola C, Gonçalves V, Sousa A, Cardoso J. Distribution and consumption of fluorite and translucent beads in the Iberian peninsula from 6 th to 2 nd millennia BC* Distribución y consumo de cuentas de fluorita y translúcidas en la península ibérica del VI al II milenios ANE. Trabajos de Prehistoria. 2020;22. doi:10.3989/tp.2020.12256
6. Garrido-Cordero JÁ, Odriozola CP, Sousa AC, Gonçalves VS, Cardoso JL. Shine on you crazy diamond: Symbolism and social use of fluorite ornaments in Iberia’s late prehistory. JLS. 2021;8. doi:10.2218/jls.3025

7. Villalobos García R, Odriozola CP, Delibes De Castro G, Santonja Gómez M, Pérez Martín R, Benet Jordana N, et al. Cadena operativa y análisis tecno-tipológico de los adornos prehistóricos de variscita del centro-sur-occidente de la Meseta Norte Española. Historia de una tradición artesanal. Complutum. 2018;29: 59–78. doi:10.5209/CMPL.62395
8. Villalobos García R. Análisis de las transformaciones sociales en la Prehistoria Reciente de la Meseta Norte Española (milenios VI-III cal a.C.). 2016.
9. Blanco Majado J, López Alonso MA, Edo Benaiges M, Fernandez-Turiel JL. Estudio analítico de determinación mineralógica y de composición química de las cuentas de collar de calaíta y otras materias del yacimiento de Las Peñas (Quiruelas de Vidriales, Zamora). 1995 [cited 21 Jul 2023]. Available: https://digital.csic.es/handle/10261/208196
10. GUERRERO MISA LJ. LA SIMA S-6 DEL COMPLEJO NEOLITICO DE VEREDILLA (BENAOCAZ, CADIZ). SPAL. 1992;1:
