# Supplementary material for: A supervised multiclass framework for mineral classification of Iberian beads
Source: PLoS One. 2024 Jul 10;19(7):e0302563. doi: 10.1371/journal.pone.0302563 (PMC11236108; doi:10.1371/journal.pone.0302563)
Supplement: S1 File — XRD Diffractograms used as a baseline to evaluate the results of the proof-of-concept. (PDF) [file pone.0302563.s006.pdf]

CLP_130

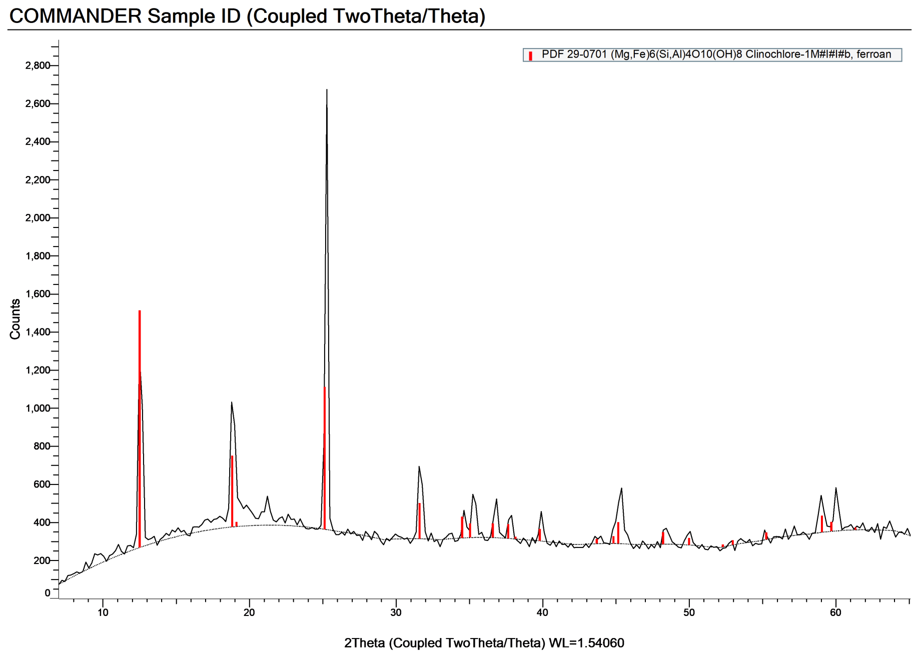

CLP_131

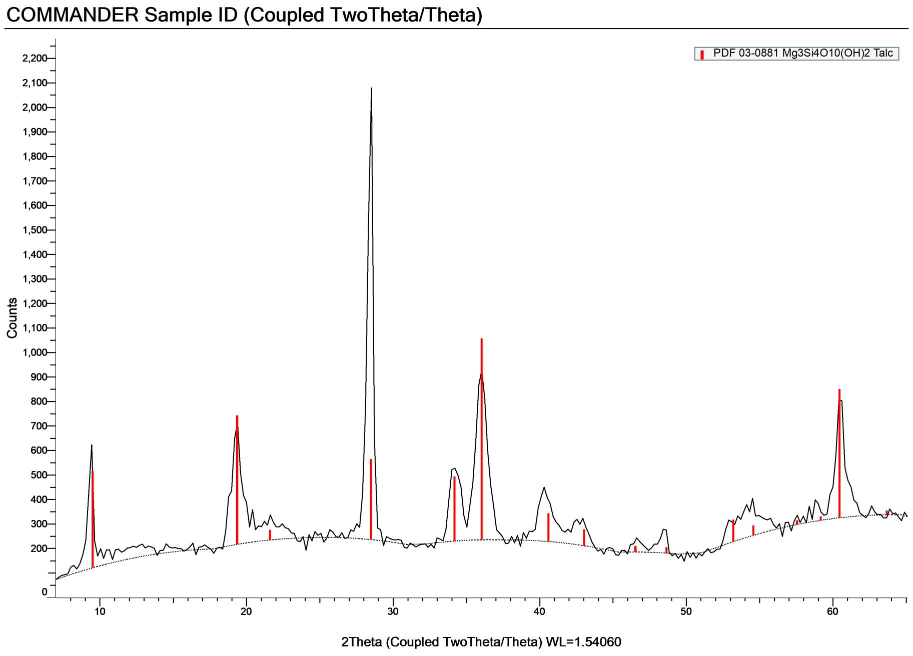

CLP_132

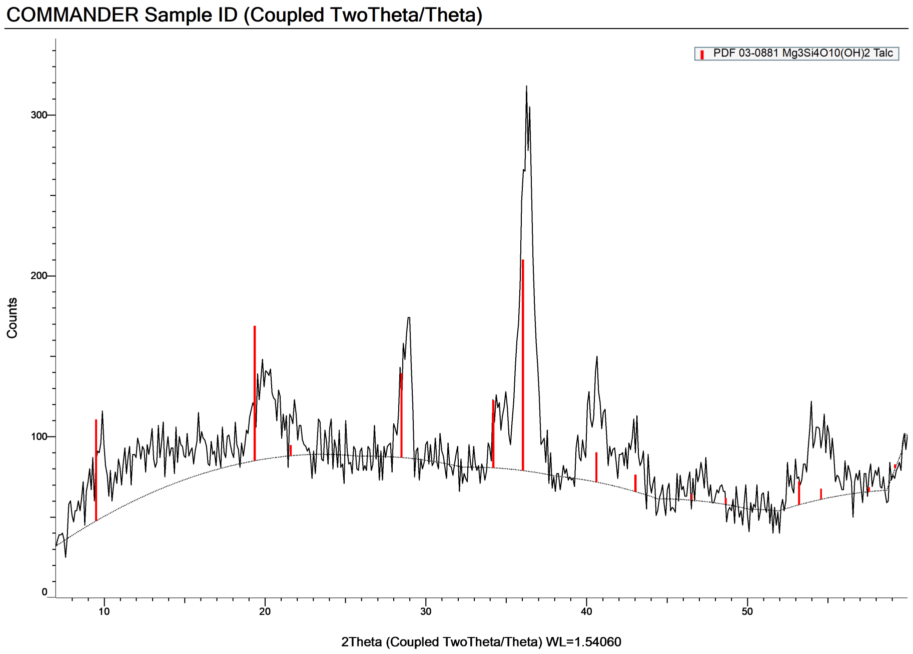

CLP_133

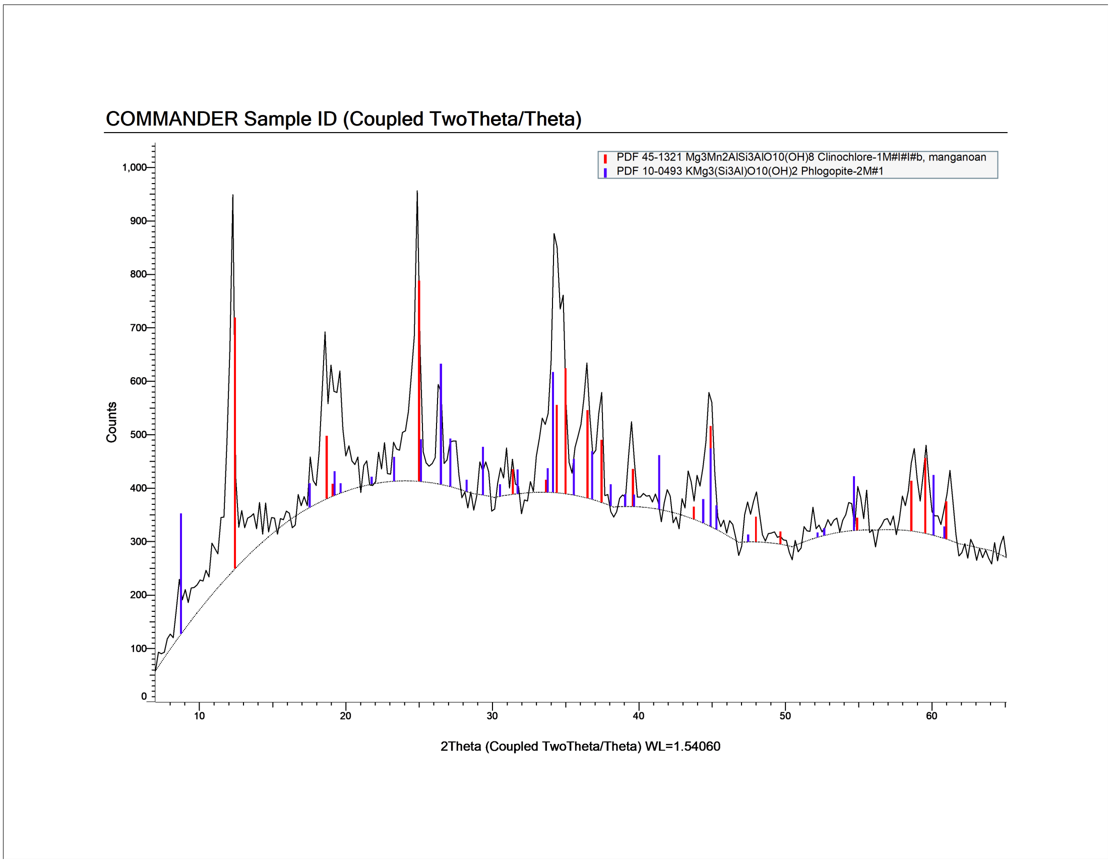

CLP_134

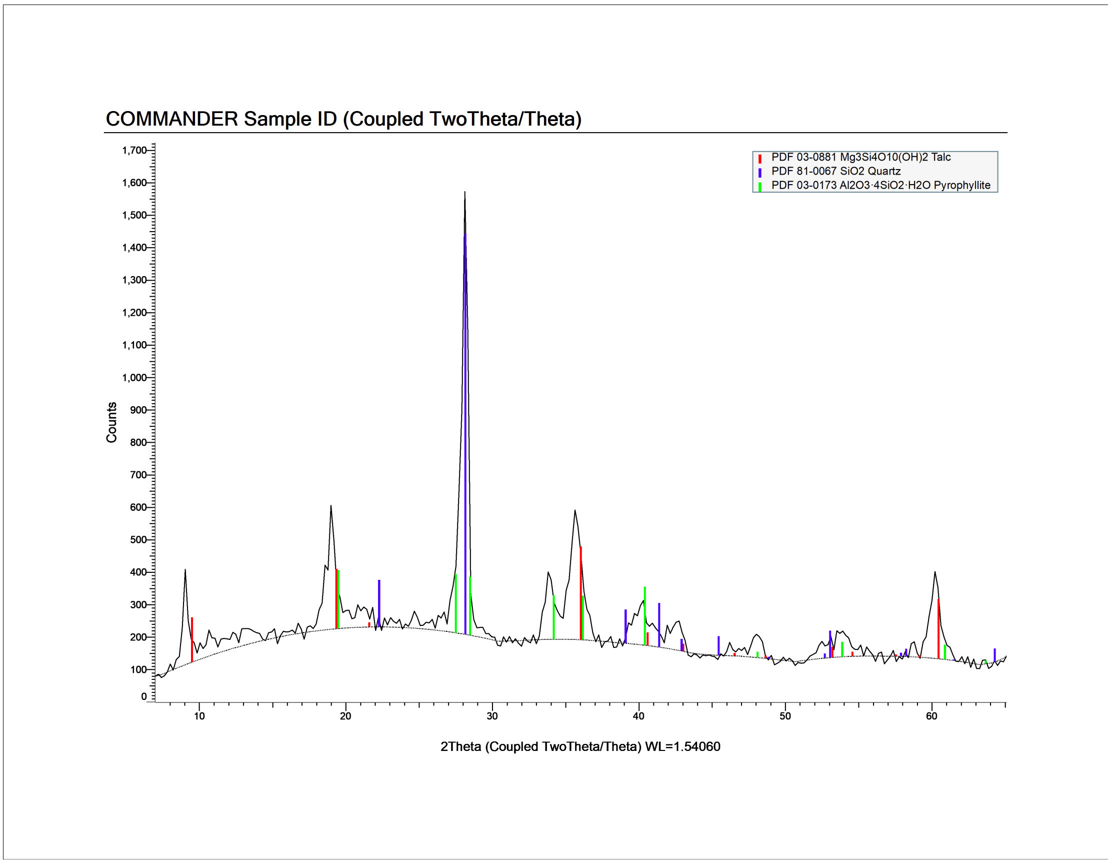

CLP_135

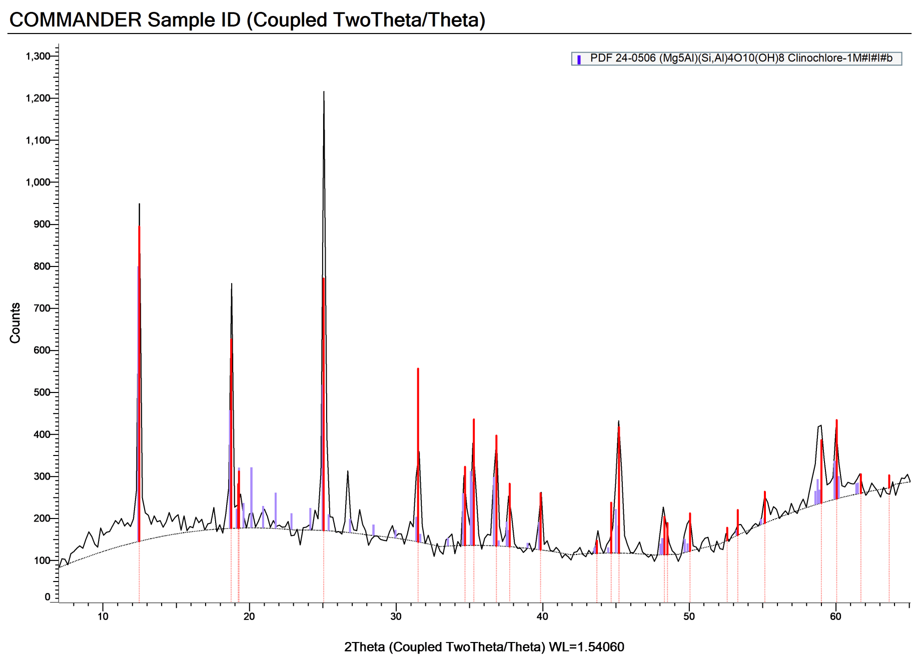

CLP_136

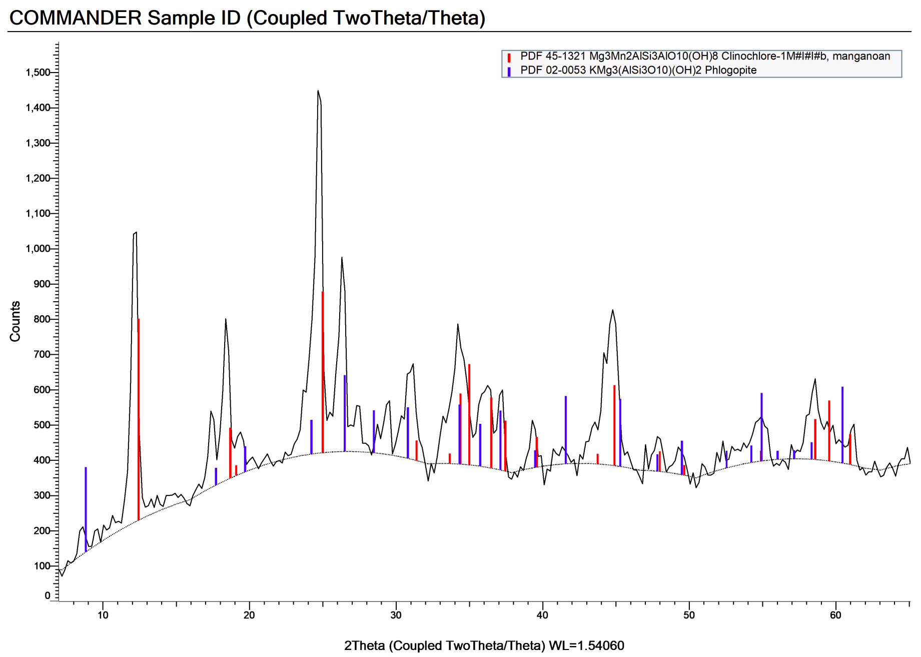

CLP_137

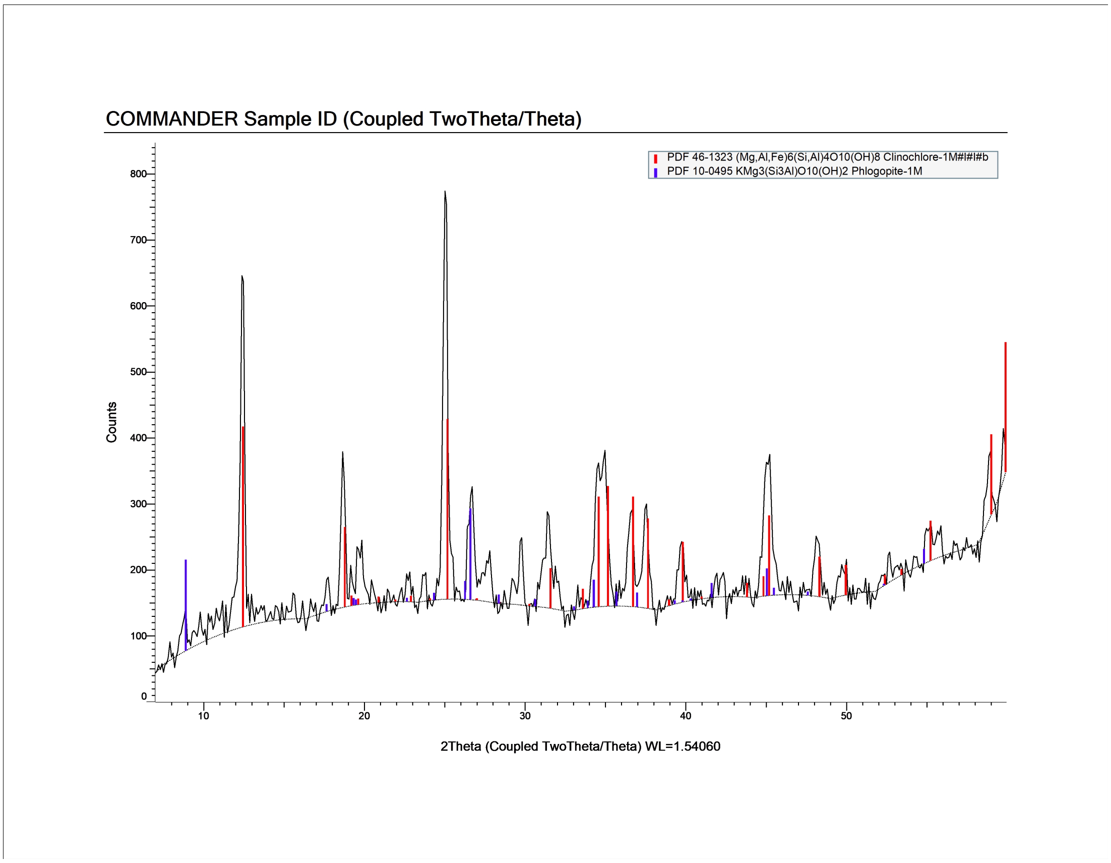

CLP_138

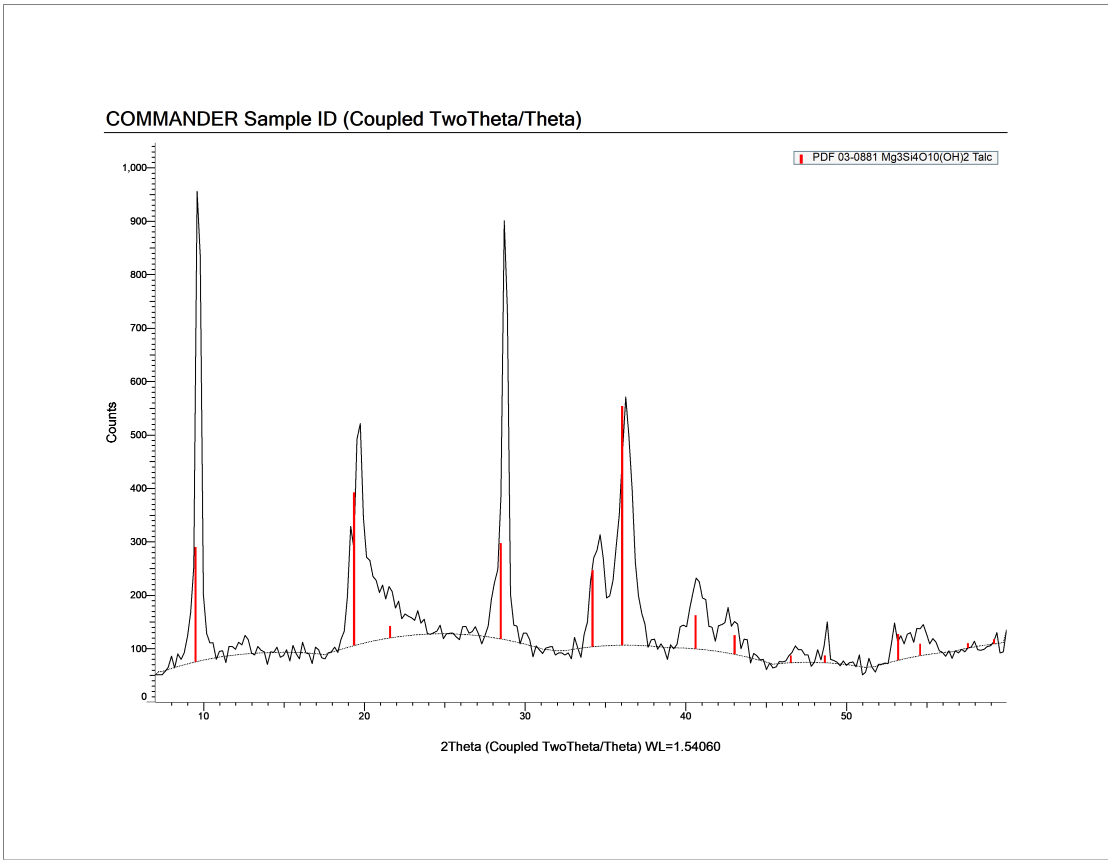

CLP_139

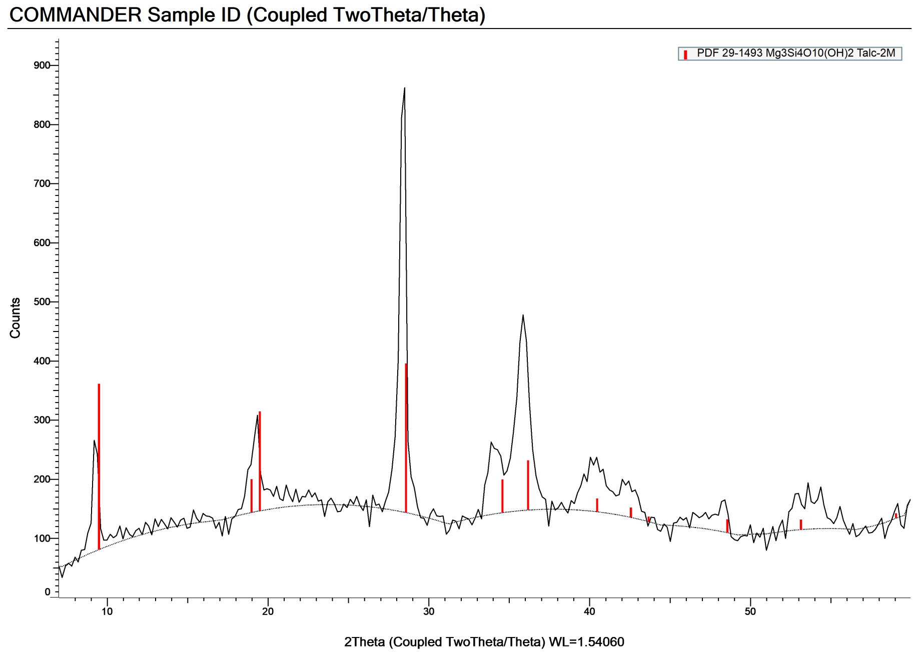

CLP_140

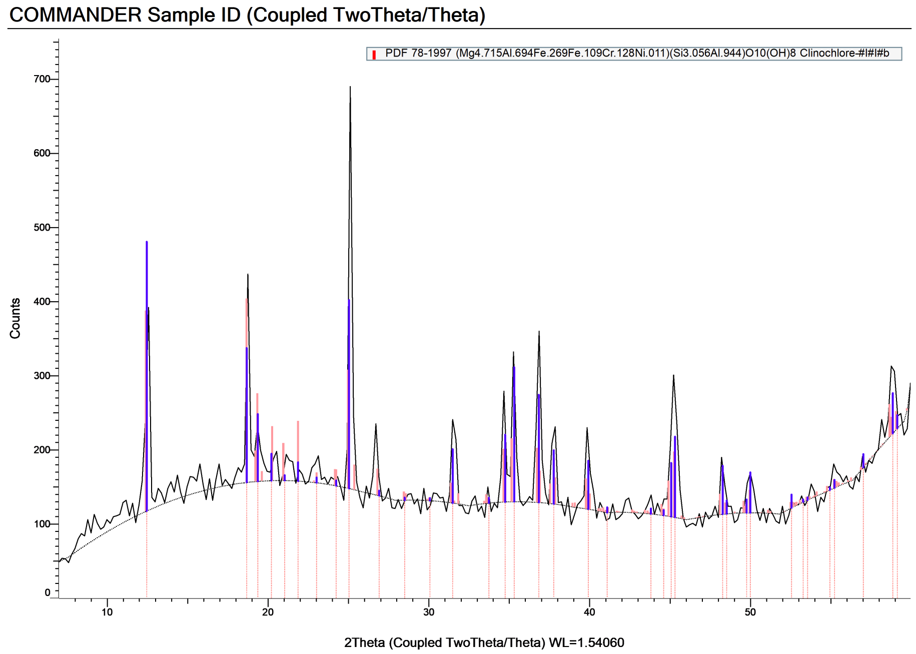

CLP_141

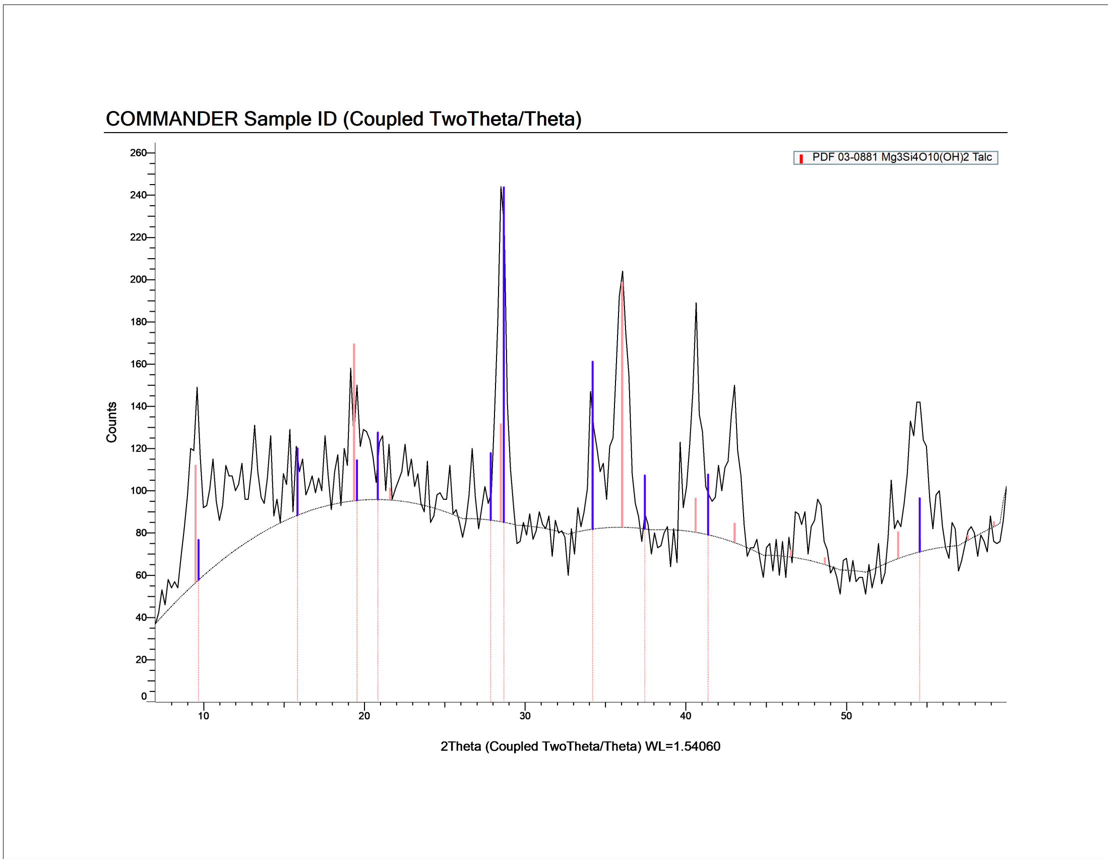

CLP_142

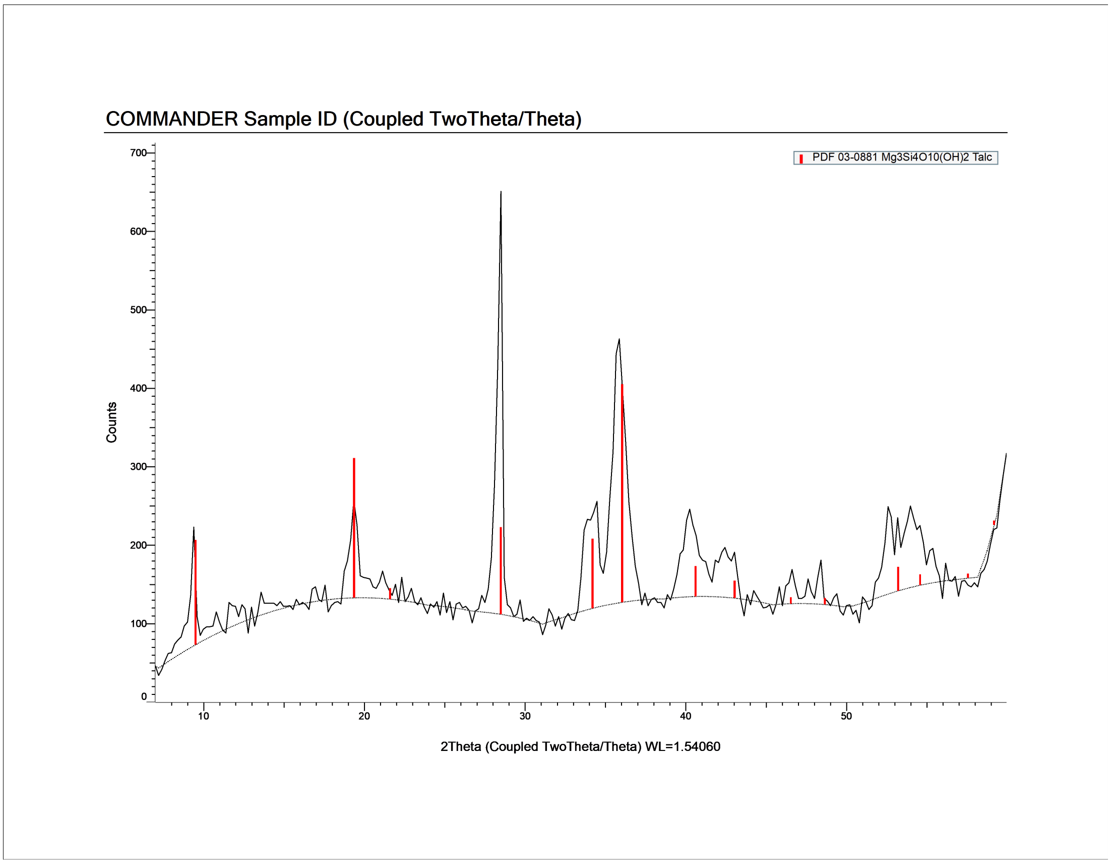

CLP_143

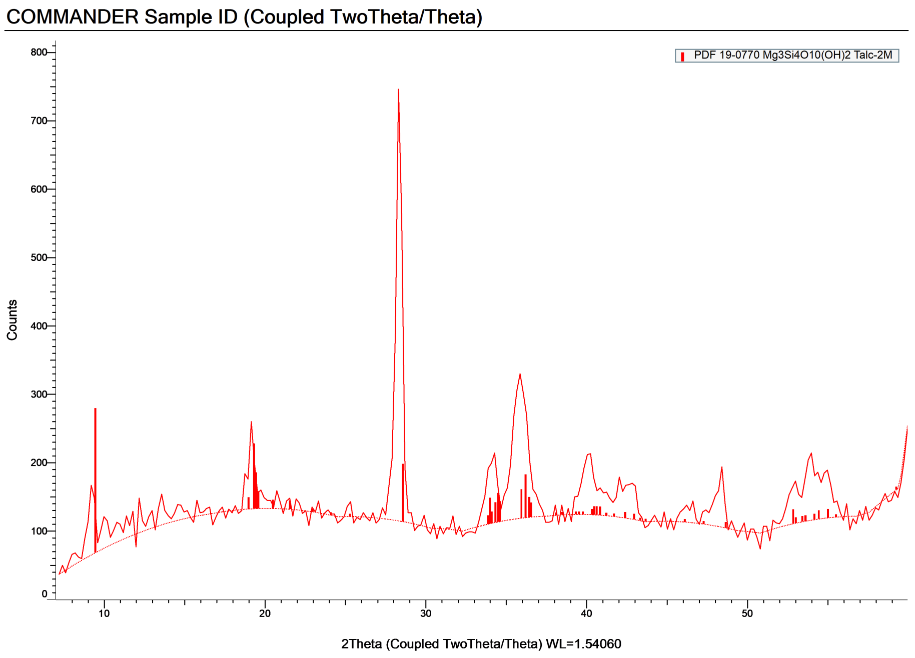

CLP_145

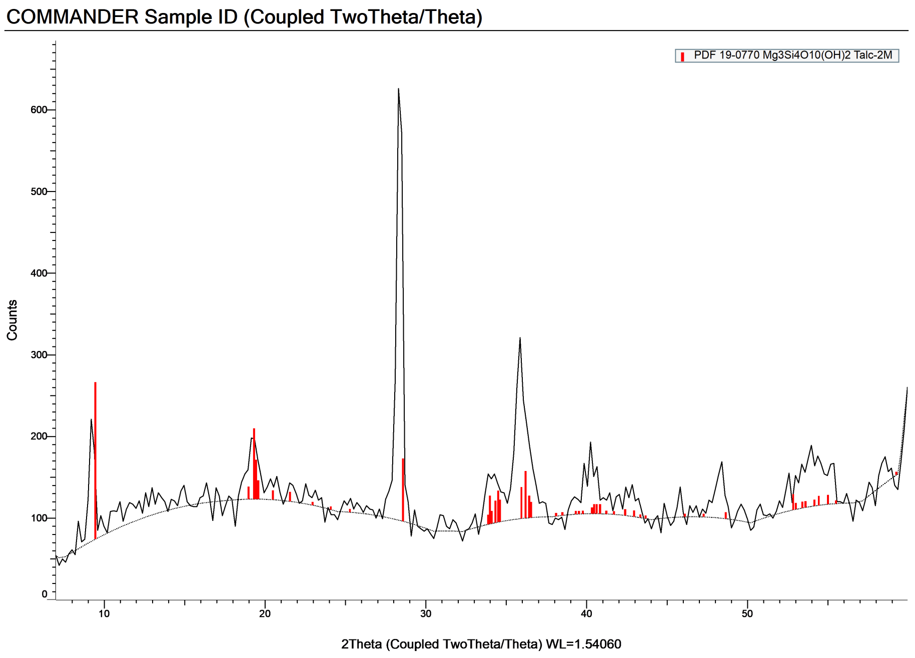

CLP_146

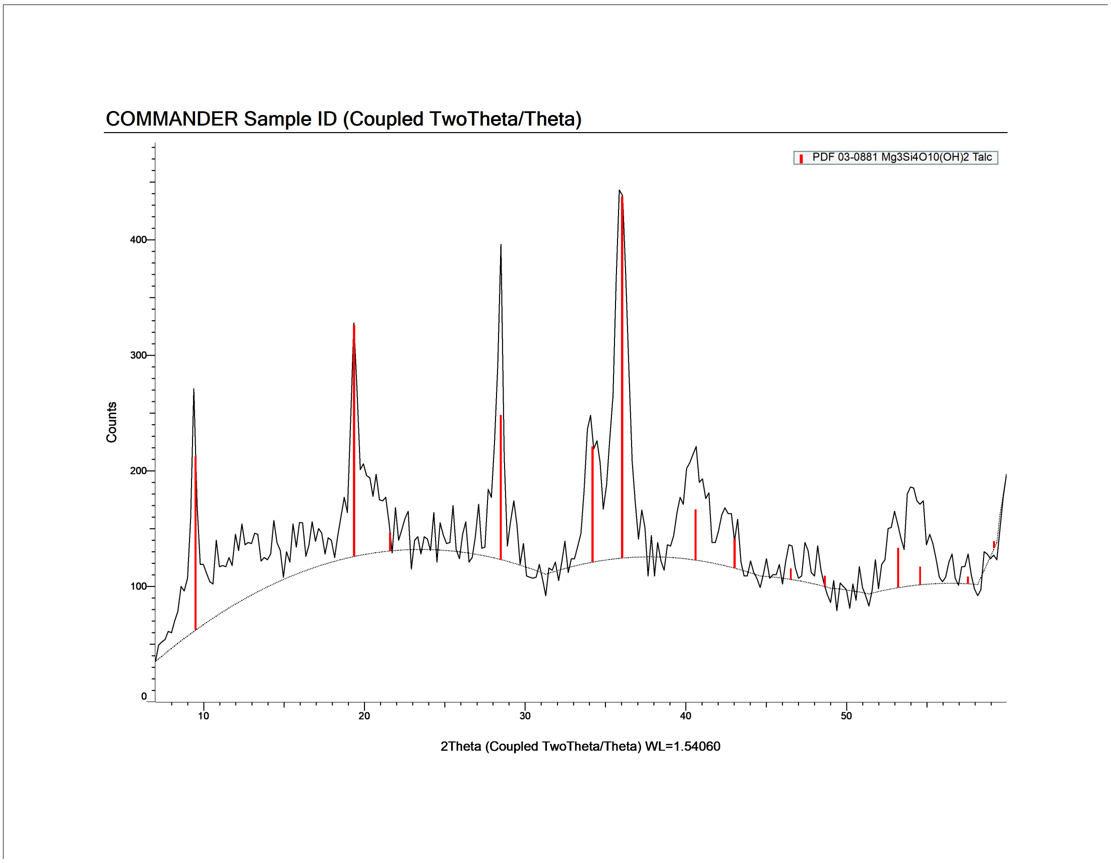

CLP_147

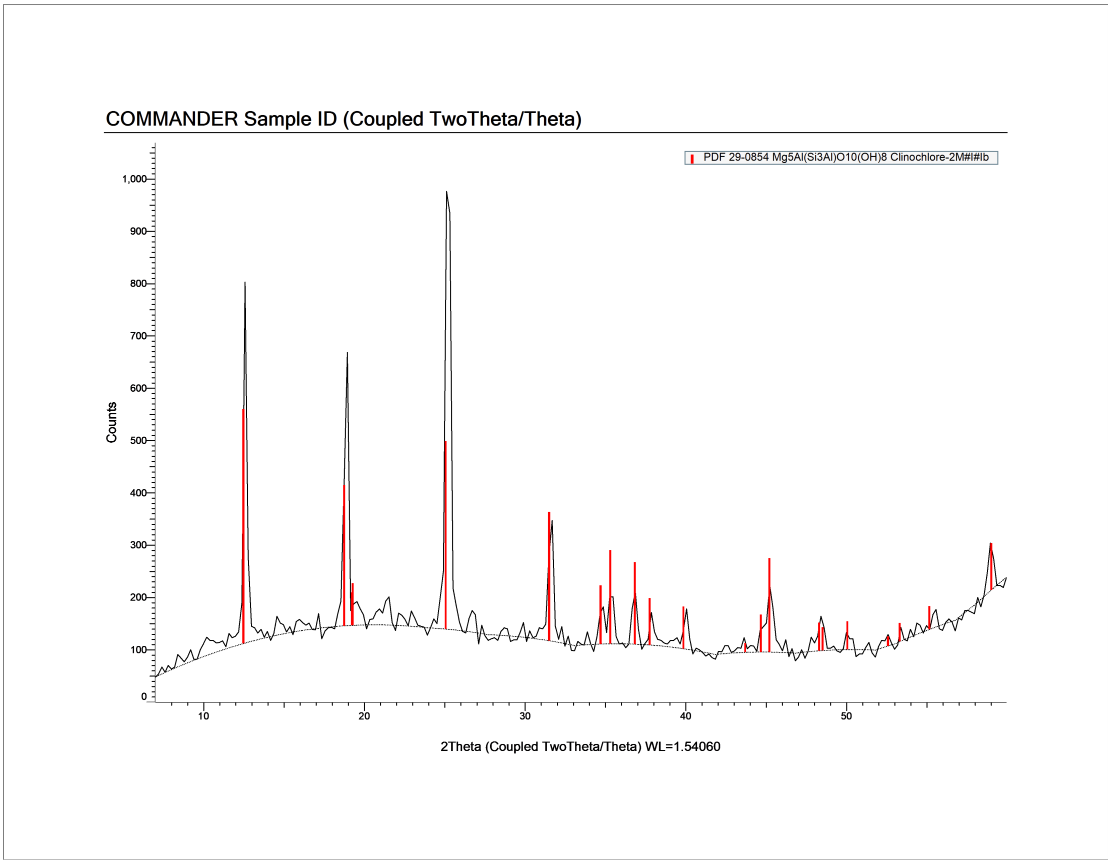

CLP_148

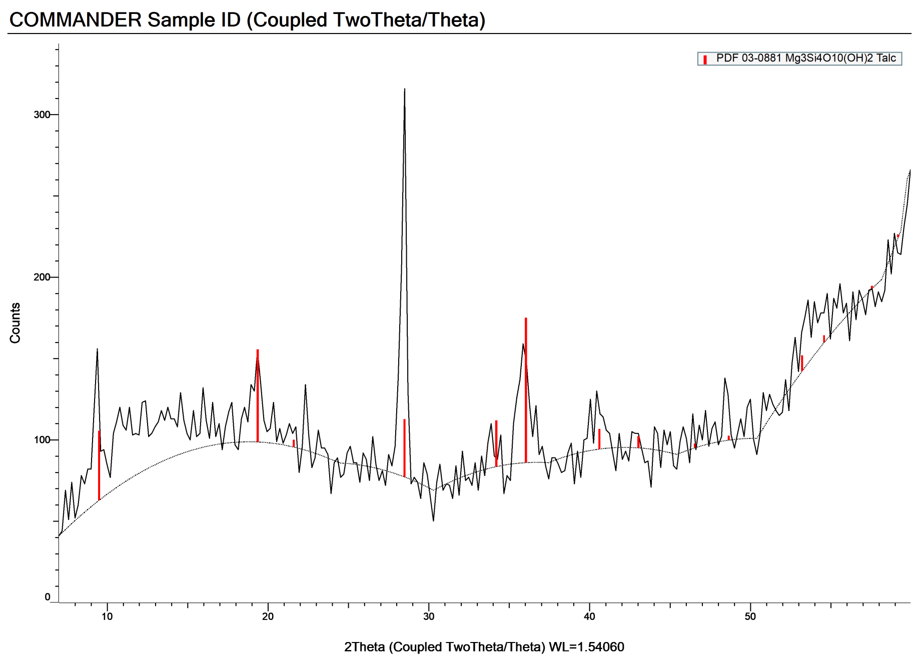

CLP_150

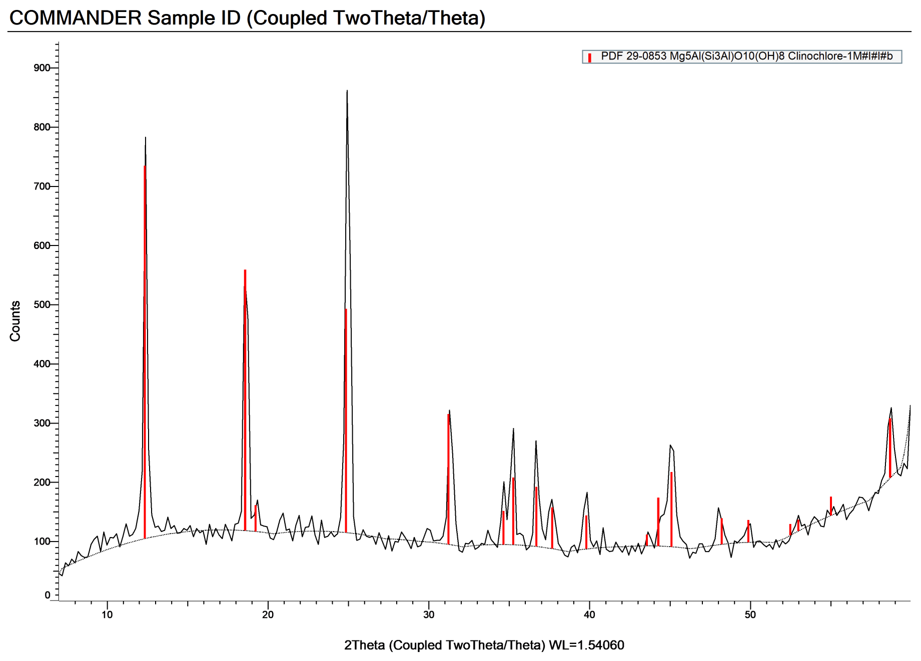

CLP_151

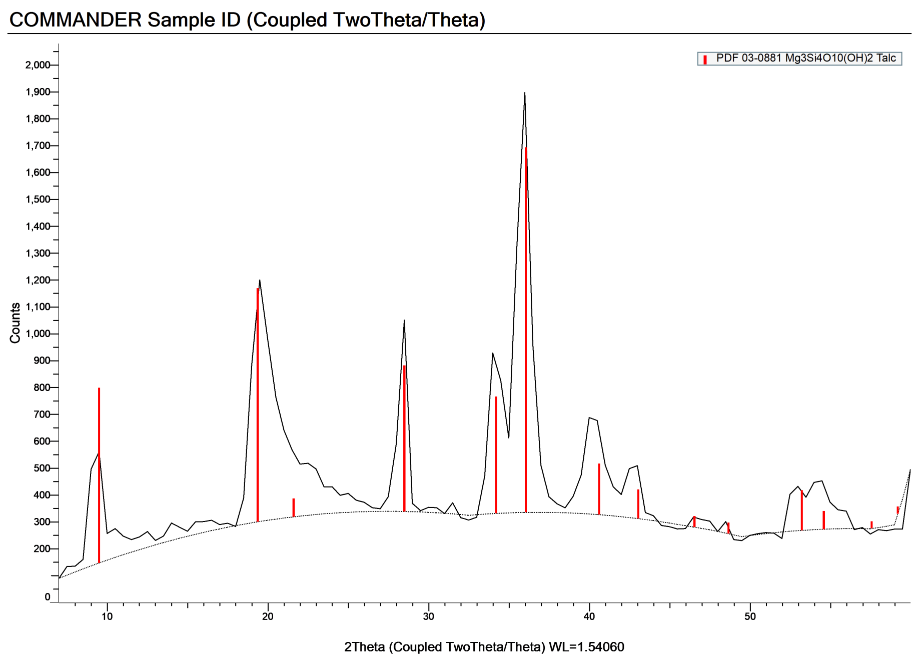

CLP_152

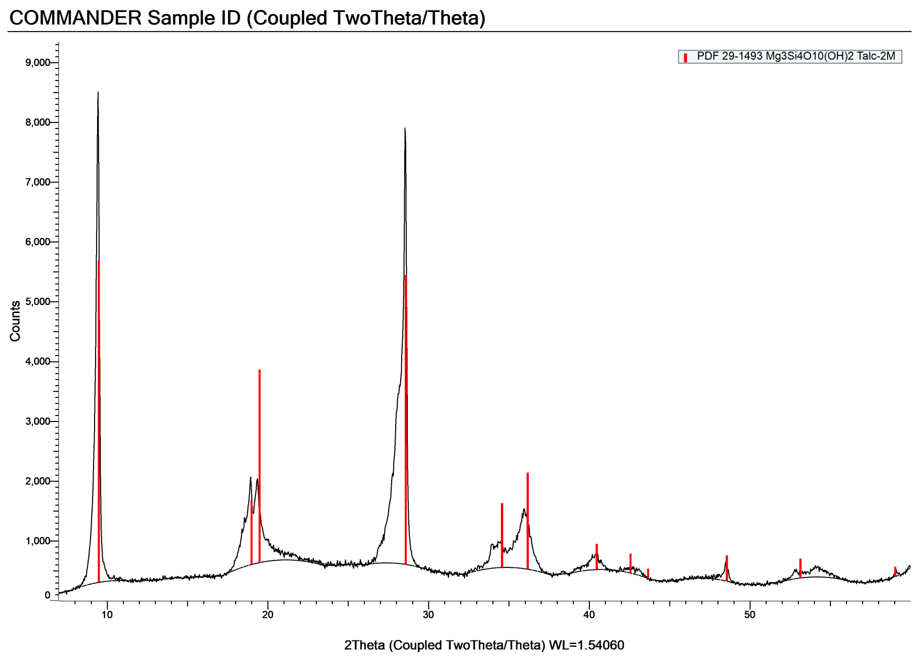

CLP_155

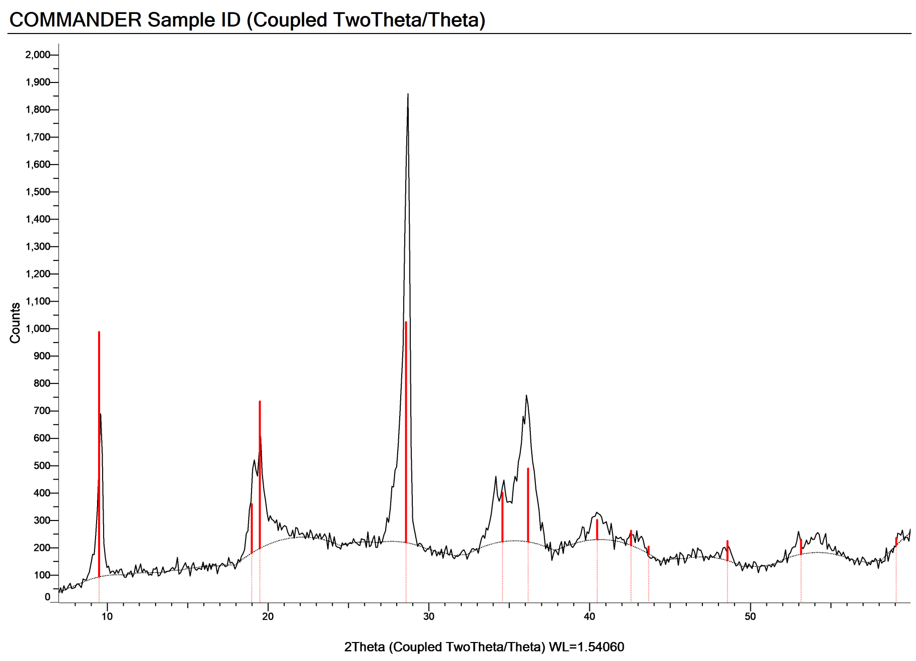

CLP_156

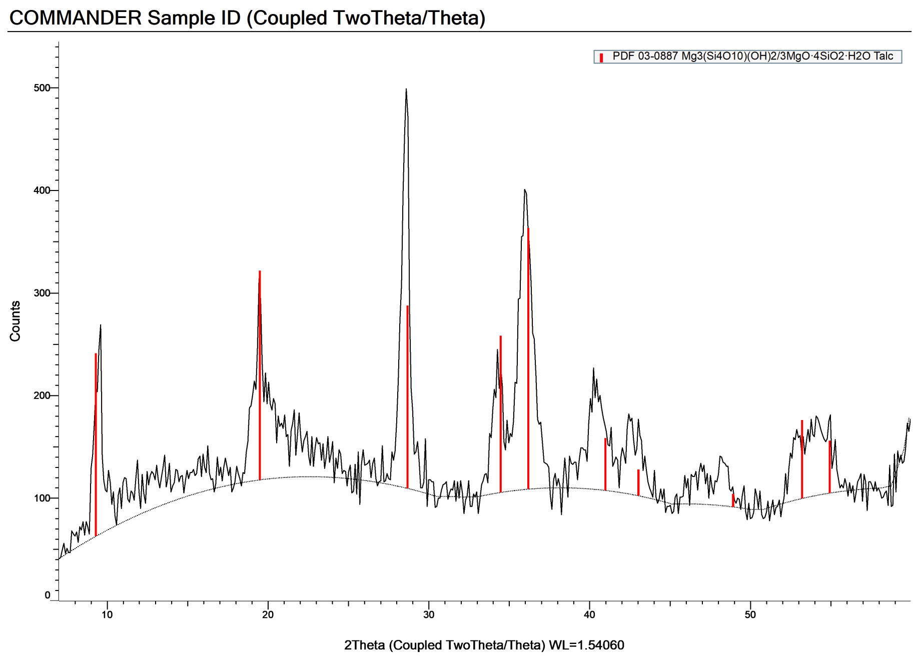

CLP_157

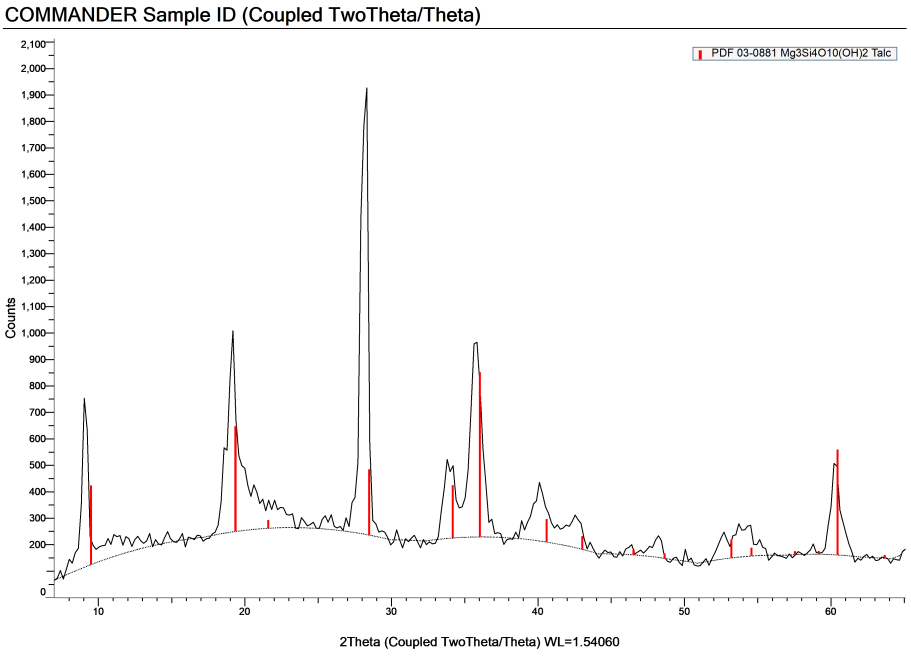

CLP_190

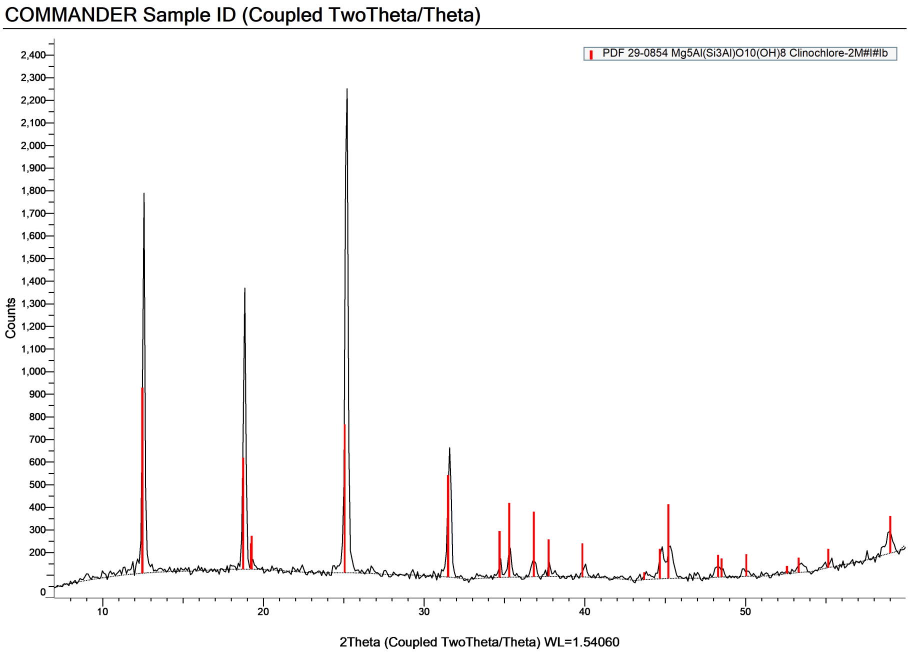

CLP_191

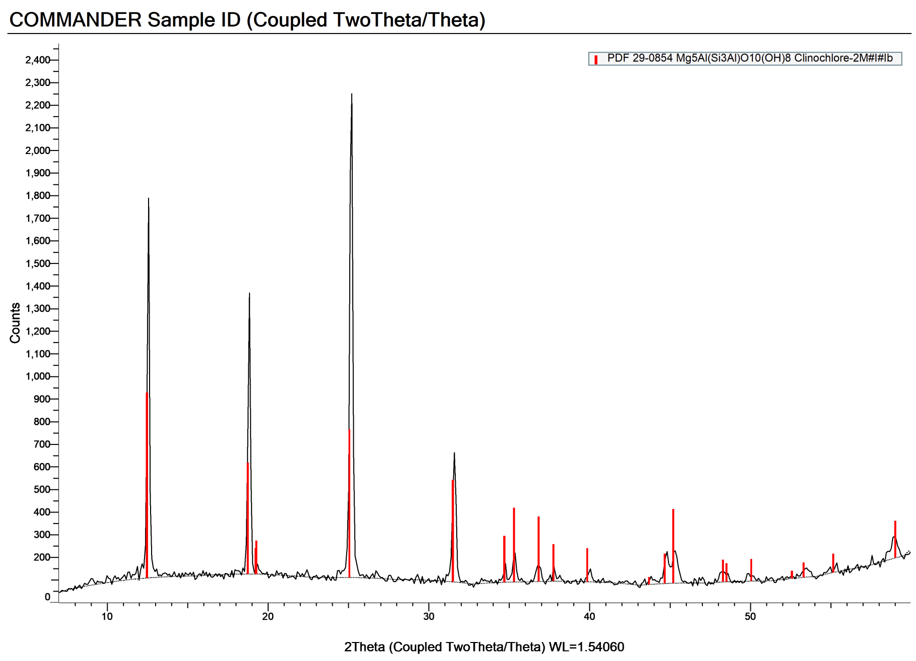
COMMANDER Sample ID (Coupled TwoTheta/Theta)
PDF 29-0854 Mg5Al(Si3Al)O10(OH)8 Clinochlore-2M#I#Ib
Counts
2,400
2,300
2,200
2,100
2,000
1,900
1,800
1,700
1,600
1,500
1,400
1,300
1,200
1,100
1,000
900
800
700
600
500
400
300
200
0
10
20
30
40
50
2Theta (Coupled TwoTheta/Theta) WL=1.54060

CLP_192

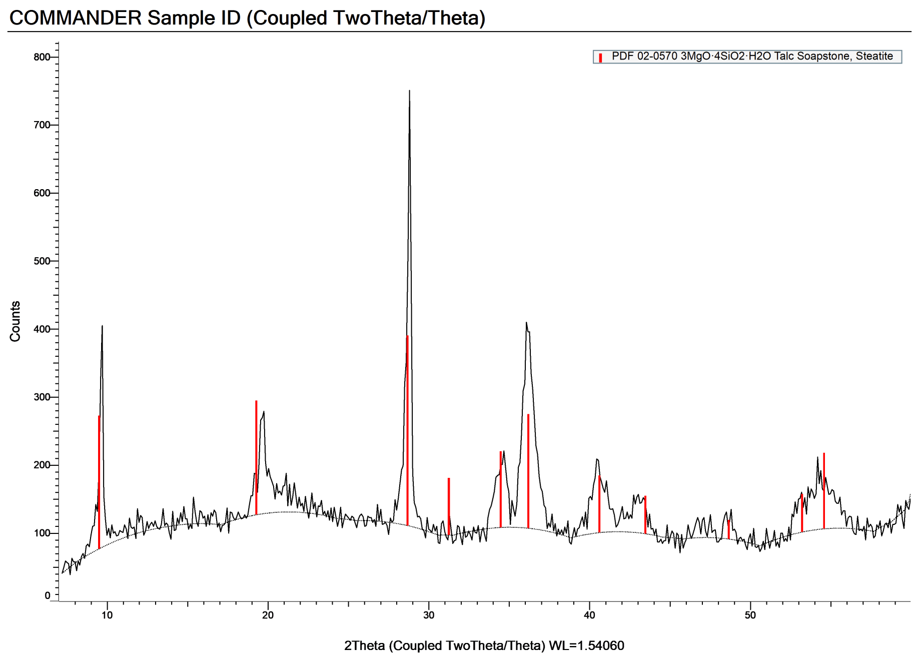
COMMANDER Sample ID (Coupled TwoTheta/Theta)
PDF 02-0570 3MgO·4SiO2·H2O Talc Soapstone, Steatite
Counts
800
700
600
500
400
300
200
100
0
10
20
30
40
50
2Theta (Coupled TwoTheta/Theta) WL=1.54060

MR(N)2

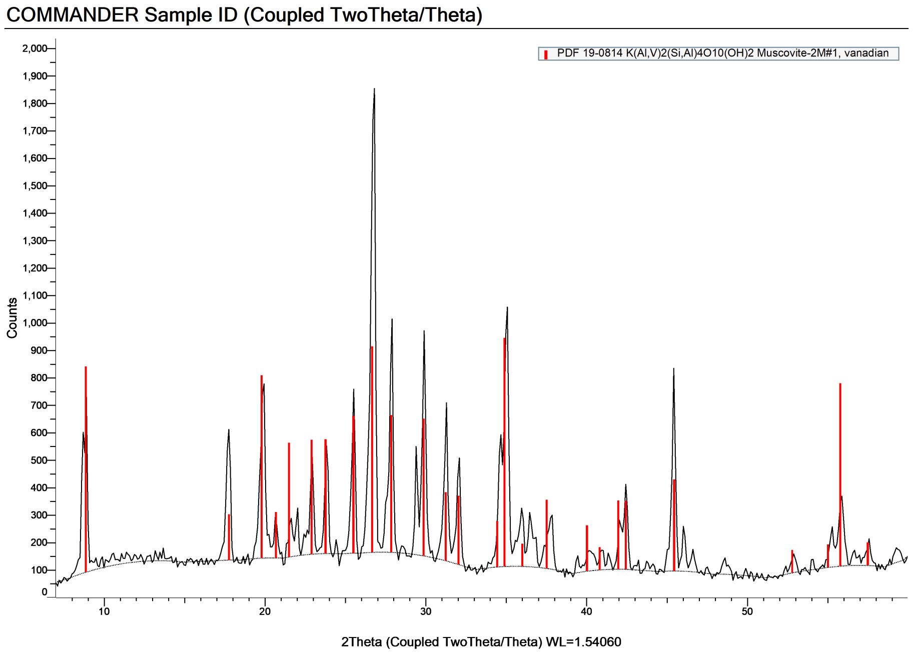
COMMANDER Sample ID (Coupled TwoTheta/Theta)
PDF 19-0814 K(Al,V)2(Si,Al)4O10(OH)2 Muscovite-2M#1, vanadian
Counts
2Theta (Coupled TwoTheta/Theta) WL=1.54060

MR(N)3

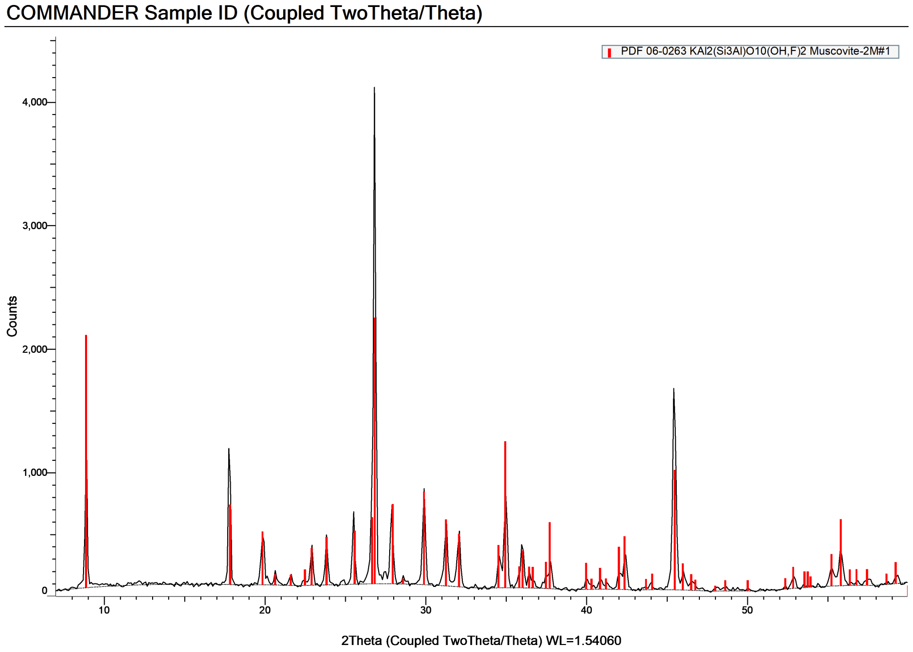
COMMANDER Sample ID (Coupled TwoTheta/Theta)
PDF 06-0263 KAl2(Si3Al)O10(OH,F)2 Muscovite-2M#1
Counts
2Theta (Coupled TwoTheta/Theta) WL=1.54060

MR(N)5

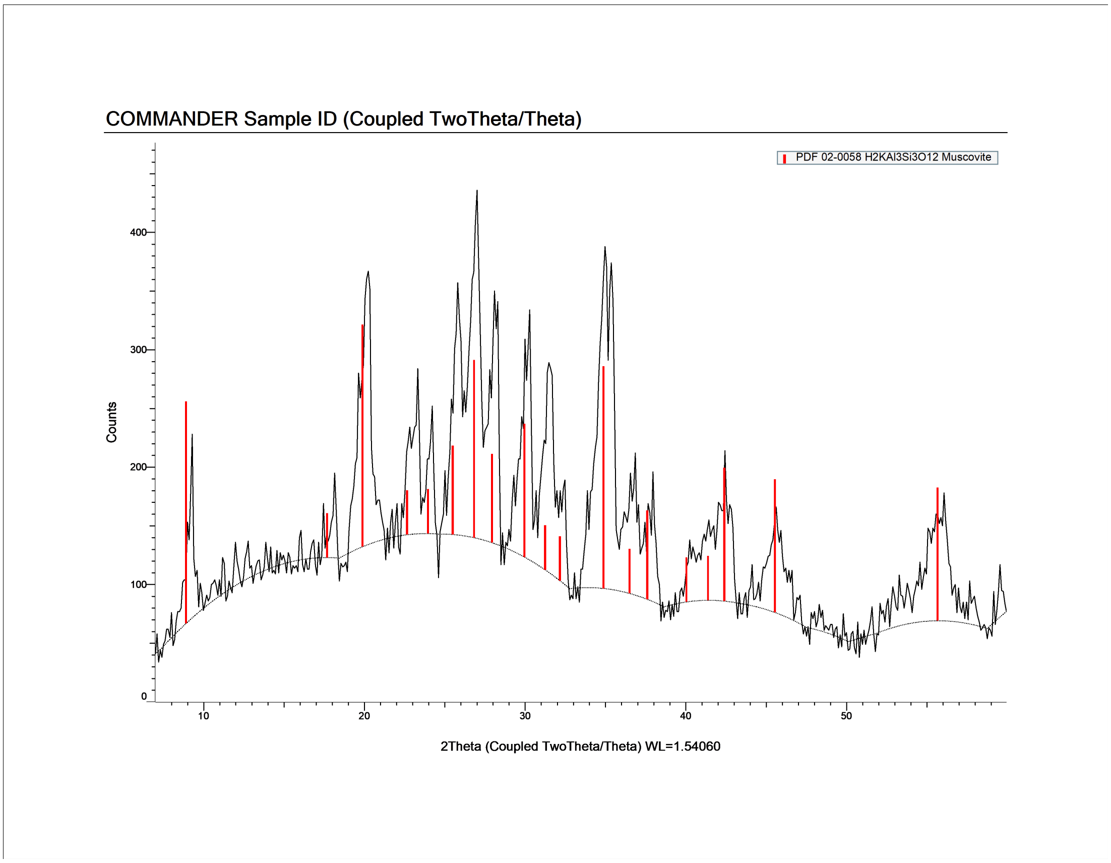

MRO_1581

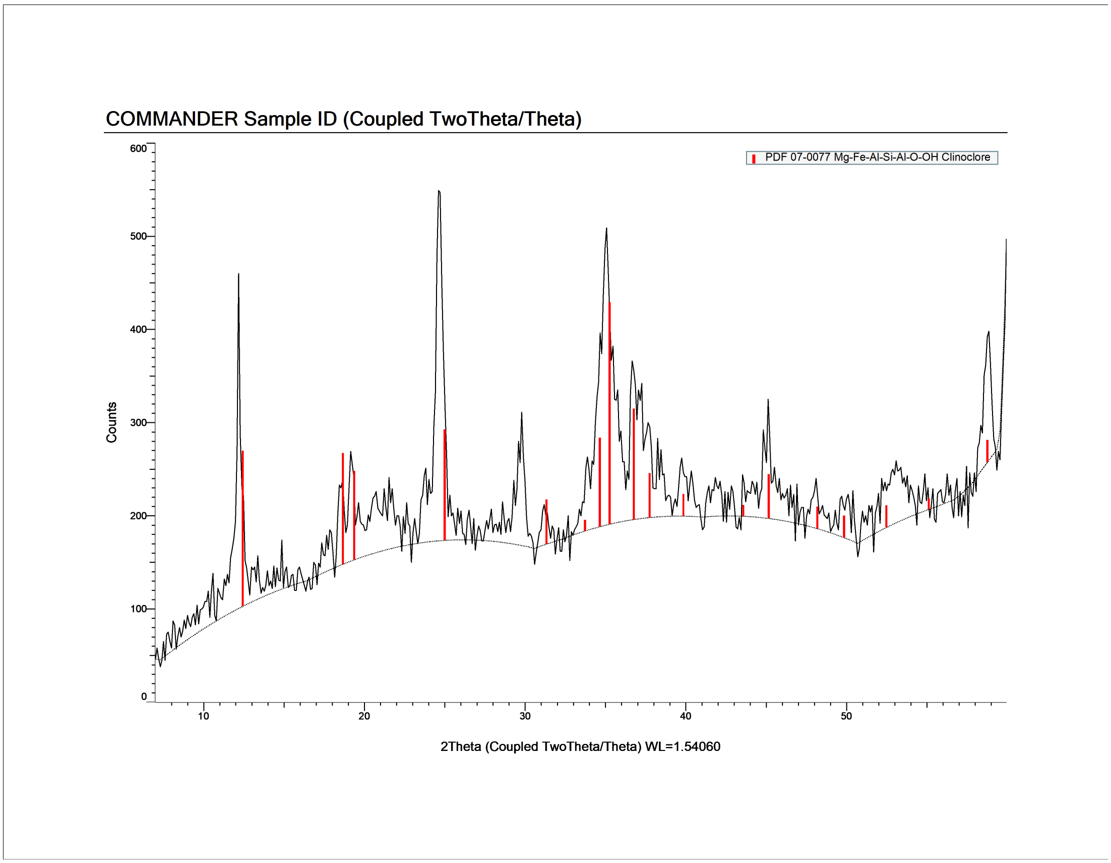

MRO_1836

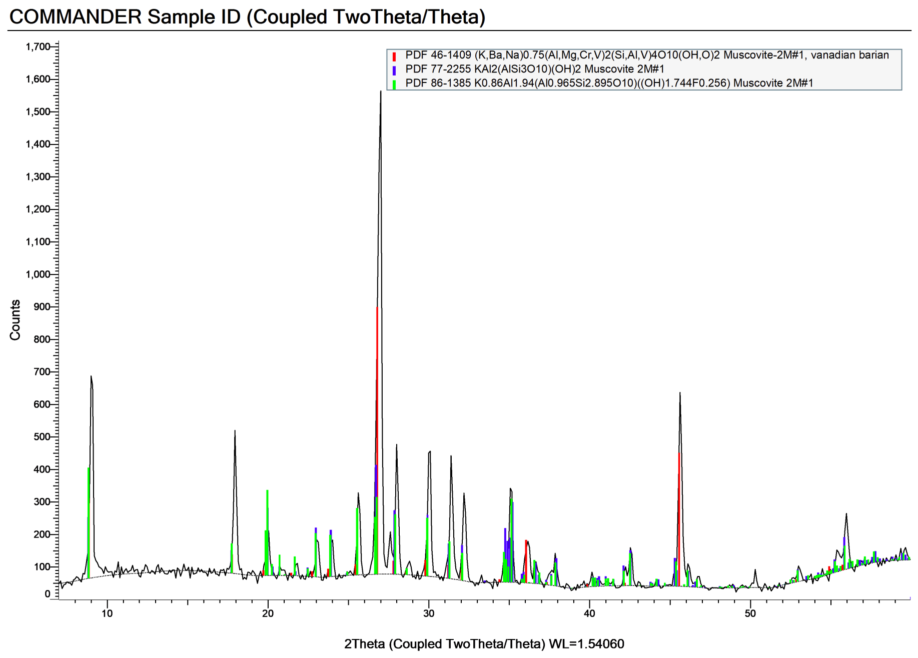

MRO_1890

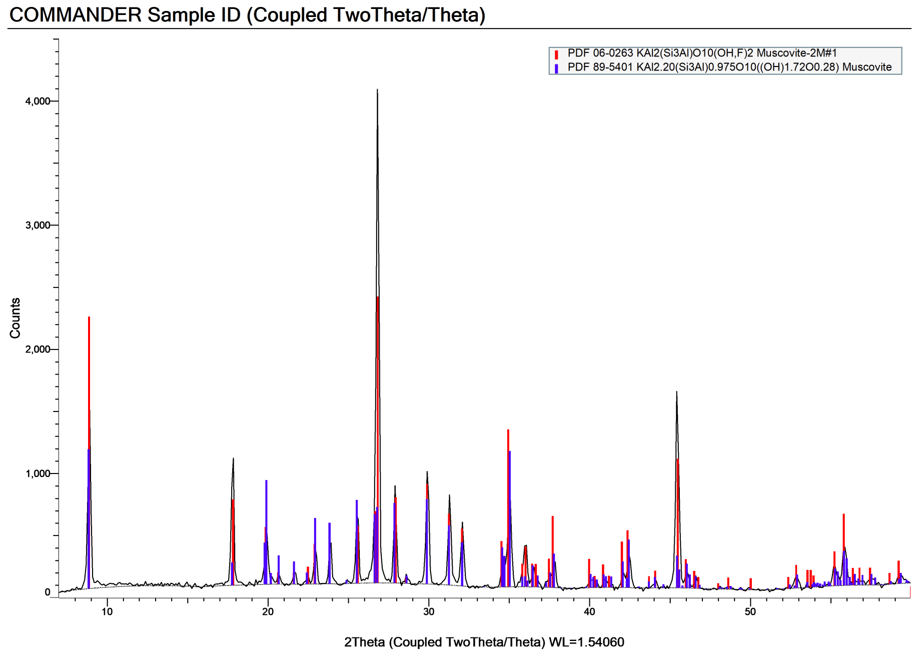

MRO_1985

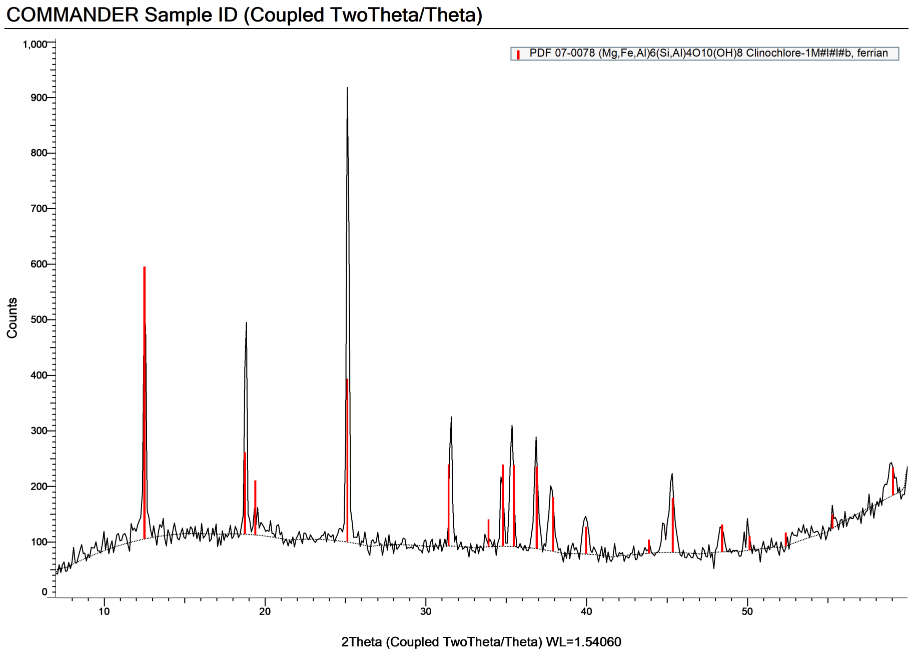
COMMANDER Sample ID (Coupled TwoTheta/Theta)
PDF 07-0078 (Mg,Fe,Al)6(Si,Al)4O10(OH)8 Clinochlore-1M#I#I#b, ferrian
Counts
1,000
900
800
700
600
500
400
300
200
100
0
10
20
30
40
50
2Theta (Coupled TwoTheta/Theta) WL=1.54060

MRO_1999

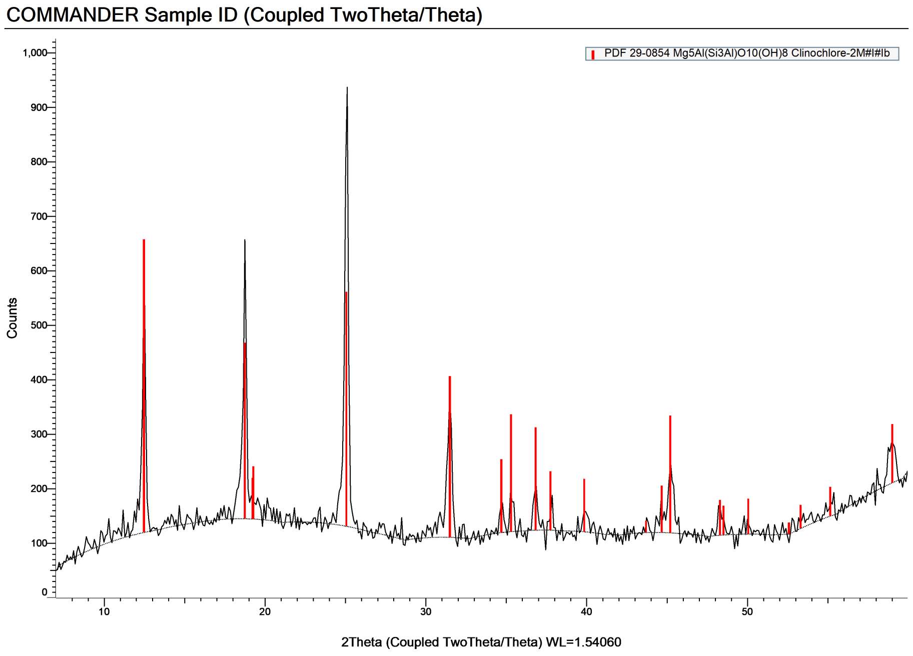
COMMANDER Sample ID (Coupled TwoTheta/Theta)
PDF 29-0854 Mg5Al(Si3Al)O10(OH)8 Clinochlore-2M#I#Ib
Counts
1,000
900
800
700
600
500
400
300
200
100
0
10
20
30
40
50
2Theta (Coupled TwoTheta/Theta) WL=1.54060

In [ ]:

In [ ]:

In [ ]:

In [ ]:
