# Supplementary material for: A supervised multiclass framework for mineral classification of Iberian beads
Source: PLoS One. 2024 Jul 10;19(7):e0302563. doi: 10.1371/journal.pone.0302563 (PMC11236108; doi:10.1371/journal.pone.0302563)
Supplement: Attachment — Submitted filename: Response_to_reviewers.pdf [file pone.0302563.s007.pdf]

We would like to acknowledge the valuable and detailed readings, recommendations and revisions made to the manuscript by both the academic editor and the reviewers. Their reviews and criticisms have been very accurate and have allowed us to improve the quality of the work. We hope that this new version will elicit a positive response from them.

We have considered all comments and addressed most of them within the revised version. In this letter, we provide responses (in blue) to each of the raised points.

Sincerely,

Daniel Sanchez-Gomez

Corresponding author (on behalf of all co-authors),

To the academic editor,

As you will see, the reviewers are generally positive about the analytical integrity of the work and your underlying technical methodology, but highlight several significant issues with the manuscript in its current form. One of their most substantial concerns is the applicability of this research both in terms of extracting social meaning with regard to your own dataset and with regard to the further applications of the method and model developed herein. In particular, Reviewer 1 draws attention to your own uncertainty about what new insights the method has brought in terms of archaeological interpretation of the dataset. This calls for a substantial reconsideration of how you present your analyses, results and their wider relevance beyond this specific exercise. Sentences that Reviewer 1 highlights, but more generally the tone of the narrative, that imply your results are "wrong" may benefit from reconsidering how you frame your evaluation if you believe your results have value and are more than simply "wrong". The value in addressing archaeological questions with the aid of this model should be more coherently justified. Overall, this study has potential and a substantial reframing of its relevance and potential impact alongside addressing specific reviewer concerns will be required before reconsideration for publication.

Please pay particular attention to required change**s.** These include demonstrating how your results are meaningful and as well as rephrasing, explaining more clearly what you mean when you say that aspects of the results were wrong and the means through which this was overcome within your model.

You must also clarify the relevance and potential impact of the model as a stand alone approach. If the opinion of the analyst remains paramount to the final outcome, in which ways has the model contributed to understanding? This requires a reconsideration of the context and potential implementation of the model developed and overall, how this is presented.

The real-world utility of the model needs to be considered with regard to studies of various scales, from individual field projects to museum-based projects using larger datasets.

The datasets / spreadsheets need more complete captioning, explanation, labelling and to be more coherently integrated into the discussion of analyses (e.g. the number of objects ultimately studied).

R:

We have reworded, modified and expanded some paragraphs and renamed some sections to improve coherence and further explain the potential and relevance of the method presented. In particular we have:

- Constructed a new paragraph in the introduction section (line 72) highlighting the challenges derived from the lack of empirical data in the study of prehistoric social dynamics related to the consumption of personal adornment in Western Europe.
- In line 341 we have built a new paragraph highlighting the advantage of decision trees over other types of classificatory models for cases such as ours (classification from chemical composition)
- In line 375 we have constructed a new paragraph explaining more clearly how the results in the validation sets were evaluated, emphasising that as the data used for the model development, they are labelled and their evaluation is easy from the standpoint of the metrics considered in the study.
- In line 443 we have renamed the "proof-of-concept" section to "proof-of-concept results".
- In line 503 we have renamed the “framework limitations” section to “ framework limitations and potentials, highlighting the potential applications of the model and its upcoming integration in our recently published PEPAdb (Prehistoric Europe’s Personal Adornment Database) project.(García et al., 2023)
- In line 482 we have constructed a paragraph explaining more clearly the great time advantage of using our approach to classify personal ornaments.

Other minor changes throughout the document have provided greater consistency, highlighted the advantages of the model in projects of different scales, and clarified concerns about the term "wrong" used to describe some results.

We have also constructed an additional supplementary information file (S1.Appendix. dataset description) in response to suggestions for a more detailed description of the dataset. This document contains specific information on the location of the specimens used, as well as data and statistics on reference materials.

All requirements of the journal and the academic editor regarding copyright and permissions on specimens used have been addressed.

We have considered all the issues raised and have provided answers to each of them for both reviewers. We consider that the changes we have made have improved the quality of the text and addressed their concerns. We hope that the revised version fits their expectations better.

Journal requirements:

2. In your manuscript, please provide additional information regarding the specimens used in your study. Ensure that you have reported human remain specimen numbers and complete repository information, including museum name and geographic location.

If permits were required, please ensure that you have provided details for all permits that were obtained, including the full name of the issuing authority, and add the following statement:

'All necessary permits were obtained for the described study, which complied with all relevant regulations.'

If no permits were required, please include the following statement:

'No permits were required for the described study, which complied with all relevant regulations.'

For more information on PLOS ONE's requirements for paleontology and archeology research, see https://journals.plos.org/plosone/s/submission-guidelines#loc-paleontology-and-archaeology-research.

R:

Within the manuscript in the "Data and code availability" (line 281) we state: No human remains were used in the present study. No permits were required for the analysis of geological samples. Permits for the analyses of archaeological samples were granted by the Chief Curators and Heads of the museums listed as supporting information in (S1. Appendix. Dataset description) as well as specific information about sample location.

3. We note that the grant information you provided in the 'Funding Information' and 'Financial Disclosure' sections do not match.

When you resubmit, please ensure that you provide the correct grant numbers for the awards you received for your study in the 'Funding Information' section.

R:

We have amended the information in the corresponding section.

4. Thank you for stating the following financial disclosure:
[This research has received resources from the Fundação para a Ciência e Tecnologia de Portugal (www.fct.pt) in the framework of the doctoral grant (UI/BD/154365/2023) awarded to (DSG) and from the Spanish Ministry of Science and Technology through the research

project (PID2021-124421NB-I00) (https://investigacion.us.es/sisius/sis_proyecto.php?idproy=33567) whose PI is (CO) and the research team is composed by (JMMB), (GRG), and (JAGC). The other authors report no funding for participation in this project.].
Please state what role the funders took in the study. If the funders had no role, please state: ""The funders had no role in study design, data collection and analysis, decision to publish, or preparation of the manuscript.""
If this statement is not correct you must amend it as needed.
Please include this amended Role of Funder statement in your cover letter; we will change the online submission form on your behalf.

R:

We have amended this information in the cover letter.

5. We note that Figure 1 and 4 in your submission contain [map/satellite] images which may be copyrighted. All PLOS content is published under the Creative Commons Attribution License (CC BY 4.0), which means that the manuscript, images, and Supporting Information files will be freely available online, and any third party is permitted to access, download, copy, distribute, and use these materials in any way, even commercially, with proper attribution. For these reasons, we cannot publish previously copyrighted maps or satellite images created using proprietary data, such as Google software (Google Maps, Street View, and Earth). For more information, see our copyright guidelines: http://journals.plos.org/plosone/s/licenses-and-copyright.

R:

Figure 1 and 4 base maps are from Natural Earth (public domain):

https://www.naturalearthdata.com/about/terms-of-use/

“All versions of Natural Earth raster + vector map data found on this website are in the public domain. You may use the maps in any manner, including modifying the content and design, electronic dissemination, and offset printing. The primary authors, Tom Patterson and Nathaniel Vaughn Kelso, and all other contributors renounce all financial claim to the maps and invites you to use them for personal, educational, and commercial purposes.

No permission is needed to use Natural Earth. Crediting the authors is unnecessary.

However, if you wish to cite the map data, simply use one of the following.

Short text: Made with Natural Earth.

Long text: Made with Natural Earth. Free vector and raster map data @ naturalearthdata.com.”

6. We note that Figure 2 and 3 in your submission contain copyrighted images. All PLOS content is published under the Creative Commons Attribution License (CC BY 4.0), which

means that the manuscript, images, and Supporting Information files will be freely available online, and any third party is permitted to access, download, copy, distribute, and use these materials in any way, even commercially, with proper attribution. For more information, see our copyright guidelines: http://journals.plos.org/plosone/s/licenses-and-copyright.

R:

Figure 2 has been created by means of drawio.com https://www.drawio.com

“There are no restrictions on the usage of any diagrams you generate with draw.io, including our online editor at app.diagrams.net. The diagrams you create belong to you and we grant you a license to use any of our copyrighted icons in your diagrams and for making raster and vector versions of your diagrams and parts, using draw.io, for any purpose.

Can they be used for any purpose under commercial usage conditions, for example?

- Yes

Does that extend to using the built-in icons that are copyrighted to JGraph?

- Yes”

https://www.drawio.com/doc/faq/usage-terms

For figure 3 we are the creators, both the images and the x/y graphic, so they can be freely used under the Creative Commons Attribution License (CC BY 4.0).

Academic Editor Comments:

1) If permits were required, please ensure that you have provided details for all permits that were obtained, including the full name of the issuing authority, and add the following statement:
'All necessary permits were obtained for the described study, which complied with all relevant regulations.'
If no permits were required, please include the following statement:
'No permits were required for the described study, which complied with all relevant regulations.'
In your manuscript, please provide additional information regarding the specimens used in your study. Ensure that you have reported specimen numbers and complete repository information, including museum name and geographic location.
For more information on PLOS ONE's requirements for paleontology and archaeology

research, see https://journals.plos.org/plosone/s/submission-guidelines#loc-paleontology-and-archaeology-research.

R:

No human remains of any kind were used in this study.

Specific Information regarding sample locations and their availability to qualified researchers is available in the supplementary material (S1.Appendix. Dataset description).

2) We note that Figures 1 and 4 in your submission contain [map/satellite] images which may be copyrighted. All PLOS content is published under the Creative Commons Attribution License (CC BY 4.0), which means that the manuscript, images, and Supporting Information files will be freely available online, and any third party is permitted to access, download, copy, distribute, and use these materials in any way, even commercially, with proper attribution. For these reasons, we cannot publish previously copyrighted maps or satellite images created using proprietary data, such as Google software (Google Maps, Street View, and Earth). For more information, see our copyright guidelines: http://journals.plos.org/plosone/s/licenses-and-copyright.

We require you to either (1) present written permission from the copyright holder to publish these figures specifically under the CC BY 4.0 license, or (2) remove the figures from your submission:

R:

Figure 1 and 4 base maps are from Natural Earth (public domain):

https://www.naturalearthdata.com/about/terms-of-use/

"All versions of Natural Earth raster + vector map data found on this website are in the public domain. You may use the maps in any manner, including modifying the content and design, electronic dissemination, and offset printing. The primary authors, Tom Patterson and Nathaniel Vaughn Kelso, and all other contributors renounce all financial claim to the maps and invites you to use them for personal, educational, and commercial purposes.

No permission is needed to use Natural Earth. Crediting the authors is unnecessary.

However, if you wish to cite the map data, simply use one of the following.

Short text: Made with Natural Earth.

Long text: Made with Natural Earth. Free vector and raster map data @ naturalearthdata.com."

a. You may seek permission from the original copyright holder of Figures 1,3,4,5,8,9 and 12

to publish the content specifically under the CC BY 4.0 license.

Reviewers' comments:

5. Review Comments to the Author

Please use the space provided to explain your answers to the questions above. You may also include additional comments for the author, including concerns about dual publication, research ethics, or publication ethics. (Please upload your review as an attachment if it exceeds 20,000 characters)

Reviewer #1: The main aim of the study presented in this manuscript is to use machine-learning algorithms to achieve mineralogical classifications of prehistoric beads. The use of this computational technique is presented as suitable in cases where the application of analytical methods other than portable XRF is not feasible. In general, the aim and execution of the exercise is original and potentially innovative, as it presents a new way to look at the classification of beads in terms of mineralogical composition. However, there remain serious doubts as to the overall efficiency and meaningfulness of the results.

To begin with, in page 15 of the manuscript, the authors themselves admit that "the chemical complexity of raw materials reflected in compositional data tends to form overlapping clusters that make it challenging to establish clear boundaries between classes, which obscures the process of model selection with this type of logic". Later, in page 19, they go on to admit that "even if the results are strictly wrong, they are very close to an accurate characterization of the samples". This is a remarkable statement in a number of ways. The authors claim that their results are wrong. The same idea is conveyed further on, in page 27: "Thus, though strictly wrong, the prediction was quite accurate".

R:

We would like to acknowledge the reviewer's careful reading of our manuscript. The wise questions and points highlighted have made us pay more attention to the way we present the details of our research and the proposed method. We have paid special attention to the terms used to present the results, which has contributed to a substantial improvement of the manuscript.

It is worth noting that on page 15, in the "model benchmarking section", we are describing the classification logic of the nearest neighbour algorithm (Knn) in comparison with the decision making process followed by decision-trees based ones, precisely to highlight the great performance of the latter given the nature of our case study. It has been a relevant result to find that even relatively simple, decision-trees are remarkably effective in solving cases such as ours (classification from chemical composition), given the performance

achieved in addition to its transparency in the interpretation of predictions as well as their low computational cost.

Thus, although we consider it relevant to maintain the explanation of the differences in the classification logics between decision trees and Knn, we have modified the paragraph (line 329) to contribute to a better understanding of the idea presented.

Before:

> Knn also showed remarkable performance and is suitable for optimization as it is possible to control the number of neighbours in the model space by which the algorithm classifies each case. However, the chemical complexity of raw materials reflected in compositional data tends to form overlapping clusters that make it challenging to establish clear boundaries between classes, which obscures the process of model selection with this type of logic. Models with other classification logics do not seem to fit the data structure and the nature of the problem as well.

After:

> Other models such as Knn also showed remarkable performance and are suitable for optimization as it is possible to control the number of neighbours in the model space by which the algorithm classifies each case. However, the chemical complexity of raw materials reflected in compositional data tends to form overlapping clusters that make it challenging to establish clear boundaries between classes when distance (e.g euclidean) is used as classification criterion, which negatively impacts the performance of models with this type of logic. Models with other classification logics do not seem to fit the data structure and the nature of our case study as well. Thus, one of the most relevant results obtained corresponds to the confirmation of the great advantage of decision trees when classifying objects according to their chemical composition, since in addition to their remarkable performance, they allow a simple and transparent interpretation of their classificatory logic, in addition to their relatively low computational cost.

On page 19 we agree that the term "wrong" is unfortunate and creates a context of doubt as to the relevance and pertinence of the whole investigation. We have modified the sentences and added what we consider is a better context for explaining model prediction errors in terms of False Positives and False Negatives in the scope of the evaluation and validation sets to emphasise that prediction errors in the model development environment (FP and FN) are not necessarily wrong in terms of the mineral classification task.

Before:

Therefore, even if the results are strictly wrong, they are very close to an accurate characterization of the samples. If we assume the three cases of variscites classified as metavariscites and two as berlinites as positive results, then the overall accuracy increases slightly.

After:

If we assume the three FN of variscites misclassified as metavariscites and two as berlinites as positive results since both species belong to the same major group of phosphates and metavariscites and variscites are polymorphs with the same elemental composition, then the overall accuracy in VS1 increases slightly.

These statements raise questions as to how the results of the computational classification are assessed or verified. How do the authors know the results are wrong? No explanation is provided of the criteria by which the results are assessed. And then, if the results are wrong, what is the point of the exercise, anyway? Immediately after admitting that the results are wrong they claim that "they are very close to an accurate description of the samples" (page 19). If the authors have a pre-ordained understanding of what is the right and wrong classification, what is the point of a computerised model that provides a result deemed essentially wrong? And how do they know it is wrong?

R:

We agree that more clarity was needed in explaining the process of evaluating the results.

Also, we have reworded the paragraph in line 214 by clarifying the form of evaluation of the proof of concept using ground truth as the baseline for evaluating the model.

We have added an introductory paragraph to the "Model evaluation on validation sets" section (Page 20) which we hope will address these concerns for future readers. We have also restructured the sentence to explain in more detail how we evaluate the model results on the validation sets. It is worth noting that since the evaluation and validation sets are part of the model development data, they are labelled and therefore their evaluation is easy from the point of view of the metrics considered in the study. Furthermore, the entire development and evaluation process, with results and metrics reports are available for reproduction through the links provided in the "data and code availability" section.

(Line 375):

Prior to deployment for real-world use, the resulting model was tested on the validation sets reserved from the beginning of the process (VS1 and VS2). It should be noted that these subsets are part of the training dataset and are

> therefore labelled data whose evaluation is synthesised in the metrics considered in this study (Table 6).

The confusion stemming from these statements is further compounded in page 29, when it is claimed that "Ultimately, the model's predictions may nicely complement the decision-making process of labelling a sample, which is, after all, up to the analyst". A clearer definition of the intended meaning of "labelling" is also required here, but if we take it to mean "classification", then the inevitable question is: if the task of classifying the beads according the mineralogy lies basically with the analyst, what is the point of the whole computational model?

R:

We agree that a restructuring of this sentence may lead to a better understanding of the presentation of the results and agree that further explanation of how the presented method improves and assists the mineralogical classification process was necessary. We have therefore constructed a paragraph at the end of the "proof of concept results" section (Line 482) emphasising the advantages in terms of time and transparency in the interpretation of the results presented by the method, especially for non-specialists.

We emphasise that the advantages of the model are particularly remarkable from a time standpoint, not only because of the possibility of obtaining an immediate classification (a label), but also because of the possibility of quickly identifying those cases where the model is not sufficiently confident in its classification. This is what we mean by an uncertainty reduction approach that significantly reduces the analysis time of a dataset with unknown or partially known mineralogy.

Consequently, we have reworded the sentence on page 29 to read as follows:

Before:

> Ultimately, the model's predictions may nicely complement the decision-making process of labelling a sample, which is, after all, up to the analyst"

After:

> Ultimately, the model's predictions greatly facilitate the decision-making process when classifying a sample, allowing for a quick and easy-to-interpret inspection of the results.

A much clearer justification of the validity and worthiness of the whole exercise is required, because otherwise it might appear futile.

R:

The lack of empirical data has been constantly pointed out by several researchers as a critical bottleneck in the archaeology of personal adornment in the Iberian Peninsula. Just as an example, in the period of review of this article, new material from a different research team has been published (Rellan et al., 2023). The state of the problem remains (as the authors point out) the compositional uncertainty (of raw materials, number of objects and chronological ascription) of the personal adornment objects leaving as the only possibility the ambiguous category of green stones to classify this abundant and heterogeneous proxy.

This has meant that current models and the implementation of new approaches such as social network analysis (SNA) lack a solid empirical basis and their representativeness is questionable. We believe that the revisions made will help to better understand the validity and potential of the method presented and hope that this revised version will fit better the reviewer's expectations.

Minor corrections

Line 114: “for the identification of bead-forming minerals” sounds a bit odd, as if the minerals had an “intention” to form beads.

R:

We have modified this term throughout the manuscript because, although it has been a "liberty" in the sense of pretending to make the point that the raw materials of mineral beads follow a non-normal distribution, such as rock-forming minerals (a concept commonly used to refer to a few minerals responsible for the formation of >90% of the rocks on the planet) and that there are therefore a few mineral species responsible for a large percentage of the diversity of the raw materials of beads so the class system of the model is reliable in reflecting the mineral diversity of the Iberian beads, it is true that the term needs further explanation and, in any case, may be due to the fact that, not being native English speakers, we have not considered the broad sense of the expression.

Line 119: where it says “two sample sets of different sites” it should say “two sample sets from different sites”

Line 125: it should say “millennia”

Line 277: where it says “our problem” it should say “our case study”

Line 305: the meaning of the term “pipeline” in the context of machine-learning algorithms should be explained, as readers not familiar with these computational models will not be familiar with it.

Line 358: The whole paragraph is duplicated

Line 376. Table 7 is too long. It should be presented as supplementary data.

Line 459: “bead carving” should be “bead making”

Line 468: This paragraph should go much earlier on in the paper, in the introduction.

R:

We appreciate the detailed reading and have followed all the indications in this regard.

Reviewer #2: The study tests and explores the potentials of using a machine learning / multiclass model for the identification of minerals in beads from the Iberian Peninsula.

The text is well written. The authors present the protocols and methodology in detail, and importantly the limitations for the algorithm.

Archaeology is still in the rather early stages of adopting machine learning methods widely (compared to other fields), and in this context the study has value. However, certain aspects of the paper are not clear. I would advise reconsideration of a few aspects before publication.

Firstly, the paper mentions a database of more than 1k objects, but in reality, a much smaller sample is used for this proof-of-concept study case. I understand that the skewed sample (with ca. 800 variscite beads) needs to be adjusted for training the model. However, as of now, my impression is that the 1.2k bead database is not relevant to the resulting model, and I do not see why it should be mentioned in the paper. The authors should highlight which are (if any) the contributions of the 1.2k bead database in the success of the model. Otherwise, I would exclude this aspect altogether.

R:

We would like to thank reviewer 2 for its patient and incisive reading. We believe that her/his comments have enabled us to considerably improve the structure of the manuscript.

We have reconsidered and rephrased the sentence in line 126 with regard to the objects analysed that constitute the database. However, it should be noted that the lack of a robust empirical basis for the study of the social dynamics surrounding the consumption of such artefacts in prehistory has been constantly pointed out by researchers and remains critical for the construction of reliable provenance models and for the analysis of social networks(Odriozola et al., 2020; Rellan et al., 2023; Rodríguez-Rellán et al., 2020; Thomas, 2011). Thus We consider the dataset used to train our model (1.2k XRD+pXRF analysis) + (n=25 proof-of-concept samples) to be a resource in itself as, to the best of our knowledge, it is the largest of its kind on the Iberian Peninsula and in much of Europe. Thus, although the main objective of the study is to present a machine learning-based method, the dataset itself is a valuable resource, highlighting the contribution of many researchers by providing freely available data, as well as being essential for the reproducibility of the method.

Secondly, even though the authors present a road map for using their model, my general critique is that I would like to see suggestions for a real-life scenario where the applicability of the model by relevant researchers in the field would be realistic and timewise sensible. For example, how many such ornaments are expected to be found on a typical site? Would this number justify the use of the model (which would require a specialized person)? In the hypothetical case of an assemblage of some 30 beads from a site, wouldn't it be possible to scan them with the pXRF and gauge their mineralogy based on the compositions? Which is what is also suggested by the authors in the case of doubtful results. If this model is destined for large museum collections, could this proof-of-concept be tested against the whole 1.2k database (and include the results in the publication)?

R:

We agree that further explanation of some of the aspects mentioned would help to better understand the advantages of the model.

First, at the end of the proof-of-concept results section (page 25) we have constructed a paragraph to highlight the advantage of the proposed method in terms mainly of time saving. This improves the explanation of the challenges of acquiring quality data, particularly in the case of XRD analysis, as even when performed by highly qualified personnel the time required increases rapidly to the point where sampling becomes a bottleneck.

Secondly, we have:

- Rephrased and renamed the section "Framework limitations and potentials" explaining in more depth the scope and challenges of the presented method including its forthcoming integration into the PEPAdb (Prehistoric Europe's Personal Adornment Database) (García et al., 2023) and its potential use in a wider cultural and geographical context.
- We have restructured some sentences throughout the text to highlight the advantages of the model in terms of the ease of interpretation of the results, particularly for non-specialists. (line 392) (line 547)
- In line (554) we propose a workflow for the interpretation of the results following what we call an uncertainty reduction approach. This means that the model not only classifies a sample by assigning a label but also allows it to assess the level of confidence with which it has labelled the sample. This allows not only to quickly classify datasets but also to identify those samples that the model has classified with low confidence. While it is true that those samples identified as problematic will require further analysis, their rapid identification within a dataset is a great time saver and facilitates the systematic sampling workflow.
- The new inspection of compositional data in the case of problematic samples represents a more informed decision making scenario as the information obtained from the model results (labels + confidence level) has reduced the uncertainty of the sample mineralogy.
- Regarding the number of ornaments expected to be found in a site, it should be noted that the casuistry of sites with mineral ornamentation in the Iberian Peninsula is very diverse and their occurrence ranges from a few to hundreds or thousands. We have made some clarifications throughout the document in order to better

contextualise this aspect and the efforts required to estimate the exact number of ornamental objects held in museums, academic centres or which form part of the reports of ongoing excavation campaigns.

- The 1.2k database used to train the model could not be used as a proof of concept as the model would perform perfectly as this is the same training data. The few cases used as proof of concept reflect the difficulty of acquiring quality data while representing average cases from sites with ornamental objects. It is worth noting that even though there are only a few pieces, the task of obtaining data on their mineralogy is a time-consuming one. In line 449 we have highlighted the advantage of using the model in this regard.

.

Regarding the question on the use of XRF to obtain mineralogical information from a sample, it is worth noting that the XRF technique does not allow the acquisition of molecular information from the sample, therefore it is impossible to obtain a specific mineral identification from this technique alone. It is possible to interpret the compositional information to establish a general classification between broad groups such as phosphates or silicates but this requires careful interpretation of the data and in any case limits the level of resolution that we intend to achieve with the presented model and that is necessary to trace the sources of extraction of raw materials.

Finally, it is not clear if this model is trained only for the Iberian Peninsula and / or only for this type of objects. Would the model be able to identify an unknown material (insteady of mislabelling it)? Please add specific information about the spectrum of applicability of this ML trained model.

R:

Our model has been trained on geoarchaeological samples from the Iberian Peninsula. That is, with objects of personal adornment and samples from the main known sources in the case of variscites. Given that the distribution of this type of objects in prehistory goes beyond the limits of the Iberian Peninsula, their scope is not geographically restricted. However, the limitation is given by the type of raw materials that make up the class system of the model, containing so far 18 mineral classes with sufficient occurrences (>10 cases per class). Thus the model will assign a label within its class system to any artefact presented, although its confidence could be quite low.

Mineral species such as jade, lapis lazuli or carnelian, etc. are not present in the model even though they represent raw materials of common use in other regions of the planet and in other periods of prehistory. It is worth noting that the model itself is ready to be reused (considering its limitations) but furthermore, the whole pipeline and the resources used for its training are freely available (including the training data) so it is expected that the p-xrf + XRD + ML method will be used to train other models adjusted to different regional problems with its respective training data.

In the section “framework limitations and potentials” we have included a new paragraph (line 570) that expands the context and utility of the presented method in other geographic regions with similar problems.

Additional comments on supplementary data provided:

S1. Database:

1. Each tab needs a table caption explaining the variables and info included in the tab. Indicate if elements are presented as such or as compounds (considering the analysis of minerals) and justify your choice.

R:

We have constructed a dataset description document presented as supplementary material (S1.Appendix.Dataset description) with detailed information about sample location, feature description and tab captions.

We have also added some tabs in the dataset with:

- XRF raw_data
- LOD and BDL imputation values
- Reference Materials data and statistics

Concerning the choice between presenting data in the form of compounds or elements, we have opted to leverage the instrument's overarching outputs by representing the data in terms of atomic percentages. This decision is underpinned by its facilitation of straightforward calculations of atomic ratios and stoichiometry. From the perspective of machine learning (ML) modelling, adopting atomic percentages has afforded us the opportunity to construct transparent models, aiding in the interpretation of results. Furthermore, this approach holds the potential, albeit speculative, to differentiate diagnostic elements – akin to chemical fingerprints – thereby enabling the identification of specific sources of raw material extraction.

2. Numbers are stored as text making it difficult to judge the data. Please convert to numbers.

R: We have double-checked that the data are in numerical format. We have noted that sometimes opening the files in different spreadsheet softwares can lead to undesirable behaviour in this regard. However, it should be noted that since the data used are fully integrated into the training process, and are accessible and reproducible through the links provided, the model could not be trained with data in text format, as the ML algorithms would raise error messages.

3. Values below the detection limits are not indicated. Only include the significant decimal places (pXRF will give many decimals that don't mean much).

R:

We have expanded the explanation of the values used to impute BDL values in our dataset (Line 231) as well as created a tab within the dataset with the LOD and imputation values

We have taken advantage of the revision process to modify the imputation of values below the detection level. Consequently we have changed the LoQ value to LOD/sqrt2. Although both are statistically negligible near zero values, some authors point out that this method generates less skewed distributions(Croghan, 2003). We acknowledge the opportunity to review this aspect.

4. Include the analysis of the standard materials with statistical information about their precision and accuracy, and respective detection limits.

R:

In the supplementary material S1 Appendix. Dataset description, we provide information about the dataset including descriptive statistics of the reference material while in the dataset itself we have created several tabs presenting the raw XRF training data, the raw data of the reference material BCR032 as well as its statistics.

The data relating to the reference materials GMB30612 and Fluka Aluminium Phosphate Monobasic could not be retrieved after an exhaustive search in the original archives (spreadsheets and .csv documents, some of them more than 6 years old). Because of this we have amended the corresponding section and omitted the use of those materials.
In the light of this situation, it is important to underline the relevance of the (fully reproducible) proof of concept presented. The materials used have been analysed with two completely different techniques and the result shows that the elemental composition reflected in the XRF data coincides with their mineralogy obtained by XRD (the ground truth). Given the results of the model and its level of accuracy in classifying these unseen data, the level of reliability of the calibration can be inferred. Of course, improvements can be made to the quantitative analyses to correct for matrix effects and in general to establish tighter calibration protocols, which we hope to develop further.
.

In line 199 we have referenced both the dataset and the supplementary material (appendix S1) where there is specific information on this point.

5. In the proof-of-concept tab, the xrd3 column is empty – why is that?

R:

Given that most of the cases in our dataset are composed of polymineral rocks, these (optional) fields are intentionally created in order to have the possibility to label the samples with more than one mineral species by means of the interpretation of the XRD diffractograms which sometimes allows to identify not only the main mineral phase but also secondary phases. However, since the column is completely empty, it has been removed to simplify their interpretation.

S2. Ground truth – Add text that explains the graphs.

R:

We have added an introductory paragraph to this supplementary material explaining the process of interpreting the graphs to identify the mineral composition of the samples.

As a bottom line, I would encourage the authors to add some more emphasis on the real-life applicability of this model and pay some more attention to the treatment and presentation of the database of pXRF values.

References:

Croghan, C. W. (2003). *Methods of Dealing with Values Below the Limit of Detection using SAS*. US-EPA. https://cfpub.epa.gov/si/si_public_record_report.cfm?Lab=NERL&dirEntryId=64046

García, G. R., Cordero, J. Á. G., Gómez, D. S., Martínez-Blanes, J. M., Sousa, A. C., Zambrana-Vega, M. D., Vallejo, M. D. S., Vera, D. R., García, R. V., & Lloret, C. P. O. (2023). *PEPAdb* [Software]. Zenodo. https://doi.org/10.5281/zenodo.8014700

Odriozola, C. P., Garrido-Cordero, J. Á., Santos, C., Barradas, E., & Sousa, A. C. (2020). The stone beads from Barrada's hypogeum 1 (Aljezur, Algarve, Portugal). Greenstone distribution patterns in the Iberian Southwest late Neolithic. *Journal of Archaeological Science: Reports*, *34*, 102667. https://doi.org/10.1016/j.jasrep.2020.102667

Rellan, C. R., Cassen, S., Carvalho, A. F., Barroso, M. M., & Valcarce, R. F. (2023). Desenredando el caos. Un análisis de la circulación de los adornos de piedra verde durante la prehistoria reciente de la península Ibérica a través de la investigación de redes. *Cuadernos de Prehistoria y Arqueología de la Universidad de Granada*, *33*,

33-70. https://doi.org/10.30827/cpag.v33i0.28274

Rodríguez-Rellán, C., Nelson, B. A., & Valcarce, R. F. (Eds.). (2020). *A Taste for Green: A global perspective on ancient jade, turquoise and variscite exchange*. Oxbow Books.

Thomas, J. T. (2011). Fashioning Identities, Forging Inequalities: Late Neolithic/Copper Age Personal Ornaments of the Portuguese Estremadura. *European Journal of Archaeology*, *14*(1-2), 29-59. https://doi.org/10.1179/146195711798369373
