# Supplementary material for: A supervised multiclass framework for mineral classification of Iberian beads
Source: PLoS One. 2024 Jul 10;19(7):e0302563. doi: 10.1371/journal.pone.0302563 (PMC11236108; doi:10.1371/journal.pone.0302563)
Supplement: Attachment — Submitted filename: Response to reviewers.docx [file pone.0302563.s008.docx]

We would like to acknowledge the constructive tone of all criticisms made by the editor and reviewers during the review process, as well as the commitment to improving the work as a whole.

We have considered all comments and addressed most of them within the revised version. In this letter, we provide responses (in blue) to each of the raised points.

Sincerely,

Daniel Sanchez-Gomez

Corresponding author (on behalf of all co-authors),

To the academic editor,

—

Thank you for your revised manuscript and for your careful attention to the comments by the reviewers and the editor of your original submission. To be accepted for publication, please reconfigure your datasheet paying close attention to the reviewer's commentary. This is essential for clarity and for accessibility. There are instances in the text where stating clearly the analytic results, including specific data, will better support your argument or require rewording of your argument to a position that is fully grounded in the data. There is capacity to demonstrate greater awareness of the limitations of portable XRF and the reviewer highlights some important elements with regard to data management, though it would be advisable to pay close attention throughout the text to issues they specifically highlight and other areas where critical awareness of those limits can make your argument more convincing. I look forward to considering a revised version of the manuscript that addresses these minor points for revision.

**R:**

We have addressed all the comments made by the editor and the reviewer, which we found to be appropriate and relevant to improve the final presentation of the paper.

Specifically we have:

- Modified the structure of the mentioned tabs within the dataset and table S2, with particular attention to formatting, clarity of information and captioning.
- Some sentences in the text have been edited or deleted to avoid excessive wording and vague affirmations that could add unnecessary noise to the discussion.
- We present the exact figures of the analyses used to train the model, avoiding vagueness.

Journal requirements:

Please review your reference list to ensure that it is complete and correct. If you have cited papers that have been retracted, please include the rationale for doing so in the manuscript text, or remove these references and replace them with relevant current references. Any changes to the reference list should be mentioned in the rebuttal letter that accompanies your revised manuscript. If you need to cite a retracted article, indicate the article’s retracted status in the References list and also include a citation and full reference for the retraction notice.

R:

Done. We have not modified the reference list.

Academic Editor Comments:

Thank you for revising your manuscript and paying close attention to the comments by both reviewers and by the editor. This version has substantially improved as a result and in most respects is suitable for publication. However, there are a few important amendments that are required before this can be accepted for publication. These primarily relate to how you present and employ your data generated with a portable XRF. I would ask you to pay close attention to the specific details of the reviewer comments and to the wider implication of these with regard to systematic presentation and clarity, supported by data, when discussing your results.

Reviewers' comments:

Review Comments to the Author

Please use the space provided to explain your answers to the questions above. You may also include additional comments for the author, including concerns about dual publication, research ethics, or publication ethics. (Please upload your review as an attachment if it exceeds 20,000 characters)

Reviewer #2: I appreciate the changes made to the text by the authors. I believe the paper in its revised form reflects more accurately the scope and aims of the study, and highlights more substantially the value of this model to the discipline. I, however, have a few additional comments for the authors to pay attention to, which I believe will strengthen the study.

Regarding S1 and S2:

1 --- Proof-of-concept tab -> S1_Database
The Proof-of-concept tab in the S1_Database file should be curated. As of now, there is what I can best describe as a raw XRF data dump. Numbers should be stored as such (they are stored text as of now, as they were with the original submission) and only the important decimals should be included, unless the authors suggest accuracy of the pXRF to 0.00000001.
For example, the value 1.5975651858007571 should be stored as 1.6. One decimal place for a value >1 % is sufficient for pXRF data and shows understanding of the technique and its limitations.

R:

Done. We have formatted the data by including a tighter decimal approximation and ensuring its numerical format.

2 --- pXRF_LOD -> S1_Database
In pXRF_LOD in S1_Dataset, please explain how you got to those LODs. From what I can understand, one CRM has been analysed: BCR32.
As an example, the authors report an LOD for Co of 0.00001. How did you get to this? This is extremely low for pXRF. This is just one example. There are more LODs that need explanation.

R:

The different approaches to calculate detection limits based on reference materials represent a considerable technical challenge for routine qualitative analysis of multi-element samples and for multianalyte methods such as XRF, as multiple reference materials for each analyte may be necessary to characterise the effects of interferences. [(Hyslop & White, 2008; Kadachi & Al-Eshaikh, 2012; Richard & Rousseau, 2001)](https://www.zotero.org/google-docs/?UoeMVd). Getting blank samples for each different composition in our dataset also requires a challenge that is not very achievable. Instead, as a standard procedure, we have used the instrument's LoD’ for a SiO2 matrix with the soil LE fundamental parameter configuration. LoD's are specified for each array at three sigma 99.7% confidence level. The individual LoD's improve as a function of the square root of the assay time. We have used LoD’s for a measurement time of 60 seconds. It is worth noting that since LoD’s are given in parts per million (ppm), we have calculated its transformation (which can be seen in the excel formulas inside the cells) to at% so that the missing value imputation fits the input format of the ML model. In the case of Co for instance, the LoD in the above mentioned configuration is 9 ppm for 60 seconds of measuring time, and its conversion to atomic percentage is 0,00002. We agree that these values are undoubtedly over-optimistic and not susceptible to be used for more specific quantitative analyses. In fact, as mentioned in the text, they are statistically negligible near-zero values. The reason for using these for the imputation of missing values instead of just zeros is that we wanted to be as accurate as possible by replacing missing values with a constant that would produce the least bias in the distribution according to the literature [(Croghan, 2003)](https://www.zotero.org/google-docs/?ZwPJsM).

On the other hand, the reference material has been used for the calibration of the apparatus in successive analysis routines, in particular for the control of the parameter values in the phosphates that make up the data set, in order to be aware of possible instrumental errors.

3 --- Ref_Material_Stats -> S1_Database
In the Ref_Material_Stats tab please show clearly the measured versus certified values (e.g. in a row just above the mean values of the 45 measurement). As of now it is impossible to judge. Also explain your LODs for the elements not included in the CRM.
Elements not detected should not be included. It is not clear to me how the elements to oxides conversion tables fit in this tab (which is dedicated to the certified reference material stats. In the same tab, report data either in wt% or in ppm. Please avoid scientific formats. This will make your data much more easy to read and will add strength to your study.

**R:**

We have addressed this issue by:

- Changing the format of the presentation of the CRM descriptive statistics, which we hope will make them easier to read.
- Added a row in the statistics table with the certified values for easy comparison with the measurements.
- Avoided scientific formats

The purpose of including the conversion of oxides to elements is because the CRM composition is reported in g/kg and we wanted to perform all the calculations within the excel sheet so that the specialised public would be aware of the reason for the values presented. Within this table are included the values of the elements in wt % and their subsequent conversion to at%.

Since the reference material is used to assess the concentrations of 9 parameters (calcium, phosphorus, carbonates, fluorine, silicon, sulphur, aluminium, magnesium and iron), we have retained all values in the calculation even though some of these elements are not detectable by the XRF instrument, however, we consider that it makes sense to include them in the calculation of the conversions, as they contain values that approximate the compositional sample to 100%.

The LoD’s used, as mentioned above, are those of the instrument and are calculated for a SiO2 matrix in the soil LE fundamental parameter configuration. They include elements not included in the reference material.

4 --- Reference_Material -> S1_Database
Why include all the elements that were not detected?

R:

We have deleted all non-detected elements from the table

5 --- S2_Table
All tables in this file need detailed captions.

R:

We have added the following description in the supplementary information section next to the file title:

This table reports the model results for the different validation subsets and for the proof of concept. The VS1_predictions and VS2_predictions tabs inform about the predictions for the two validation subsets reserved at different stages of the model development process. VS1 corresponds to a subset reserved prior to the resampling process. VS2 contains resampled (i.e. synthetic) data. The tab proof_of_concept_predictions reports the results obtained for the use case presented in this study. They correspond to data never seen by the model and are therefore assumed to be results of the actual generalisation capacity of the model.

In-text comments (revised manuscript):

- Lines 114-155: 1) A typical handheld pXRF has a standard spot area (ellipse in shape) of ca. 3 mm in its longest side. This is a large enough area to offer representative data for a bead. Unless the bead is made of minerals containing crystals and feldspars >3 mm. 2) Additionally, what does relative superficiality mean in the context of this sentence? Superficiality of analysis area? That would depend on the sample (cut samples can be analysed). Superficiality in terms of data quality? This takes me back to a reported LOD for CO that is way too low to be realistic. Overall, this sentence seems to be purposefully vague. Please revise showing a solid understanding of the evaluation of handheld pXRF analyses.

R:

The central argument of the paragraph is that the XRF technique should be complemented with other mineralogical analyses, as fluorescence is not able to reveal the molecular composition of the beads. Otherwise, we agree that the spot area (9 mm in our device) is sufficient to provide representative information on the objects analysed in this study and that the term superficiality (which referred to the depth of penetration of the method given the variability of the samples and the impossibility of homogenising them given their archaeological nature) lends itself to ambiguous interpretations. Therefore, we consider that this sentence adds unnecessary noise to the purpose of explaining the complementary use of analytical techniques to obtain a detailed compositional and mineralogical characterisation of the objects, so we have chosen to keep the original version, which did not raise any comments.

- Line 126: Replacing “10k beads” with “some thousands of beads” takes away credibility from the text. Just state how many beads you analysed and keep it simple. Avoid vagueness.

R:

We have simplified the sentence by including strictly the data used to train the model, avoiding vague and overstated statements. We hope that this clarification meets the reviewer's expectations, while providing accurate information on the database.

The sentence has therefore been amended as follows:

Before:

Over the past decades, we have analysed some thousands of beads beads by pXRF and, when possible, by XRD, creating the largest geochemical data set (compositional and mineralogical) on personal adornments from Iberia (S1 Dataset)

After:

In recent years we have created a database that to date has (n=1243) compositional and mineralogical analyses by pXRF and XRD of elements of personal adornment from the Iberian Peninsula.

- Line 133 : MS is (not are) unless Machine learning is meant to be plural here.

R:

Done

- Lines 133-138: Though not strictly wrong, this paragraph reads as rather vague (again). It would be nice to have it with more relevance to the specific study at hand.

R:

We have rephrased this sentence as follows for the purpose of greater precision:

Before:

Machine learning (ML) are reshaping archaeology by automating existing workflows of data analysis and introducing innovative approaches that can potentially reveal macro-archaeological patterns thanks to the increasing computational power, the accessibility of algorithms in addition to the vast amounts of data that are constantly emerging and because most archaeological problems can be formalised in some way as classification or regression tasks for which algorithms are particularly well suited

After:

Machine learning has become a powerful alternative in archaeology thanks to the increasing computational power, the accessibility of algorithms and the growing amount of data that is constantly emerging.

- Line 231: How and why were

R:

To address this issue, we add the following sentence:

To calculate the imputation values, we used the device's limits of detection (LoD) for a SiO2 matrix and estimated their conversion from parts per million (ppm) to atomic percentage to fit the input format of the model (S1_Dataset). Although these are near-zero values negligible from a statistical standpoint , we wanted to replace the missing values with a constant that would produce the smallest bias in the distribution according to [30].

- Line 463: A small change, nevertheless an important one: “two completely different sites” -> why not just write “two sites” and avoid unnecessary wordiness. This is an example. Similar comments apply throughout the text.

R:

Done. We have gone through the whole text again to address this situation, as a result of which we have edited or deleted some sentences that did not add content to the discussion.

- Line 563: Do you mean the archaeology of personal adornments in general or those made by minerals such as the beads? Personal adornments can be made from a variety of materials.

R:

Line 563 refers (in the revised version with active change control) to the characterisation of new cases that will help us to map the distribution and consumption patterns of personal mineral ornaments.We have slightly modified the phrase to emphasise its application to mineral ornaments and to avoid possible confusion. Consequently the sentence has been reworded as follows:

We propose a workflow for the interpretation of the results that is being used by our team for the characterisation of new cases that will help **us to map the distribution patterns of mineral adornments in the Iberian Peninsula**; i) check that the predictions at both levels match ii) check the confidence of the probability of the prediction and set a confidence threshold (we propose ~85%) to maintain the label generated by the model.

Once these comments are addressed, I am happy to see this study in print.

References:

[Croghan, C. W. (2003). *Methods of Dealing with Values Below the Limit of Detection using SAS*. US-EPA. https://cfpub.epa.gov/si/si_public_record_report.cfm?Lab=NERL&dirEntryId=64046](https://www.zotero.org/google-docs/?1CTHle)

[Hyslop, N. P., & White, W. H. (2008). An Empirical Approach to Estimating Detection Limits Using Collocated Data. *Environmental Science & Technology*, *42*(14), 5235-5240. https://doi.org/10.1021/es7025196](https://www.zotero.org/google-docs/?1CTHle)

[Kadachi, A. N., & Al-Eshaikh, M. A. (2012). Limits of detection in XRF spectroscopy. *X-Ray Spectrometry*.](https://www.zotero.org/google-docs/?1CTHle)

[Richard, D., & Rousseau, R. (2001). Detection limit and estimate of uncertainty of analytical XRF results. *THE RIGAKU JOURNAL VOL*, *18*.](https://www.zotero.org/google-docs/?1CTHle)
